# Prognostic value of peri-operative circulating tumour DNA levels estimated by cell-free DNA methylation in patients with resectable colorectal liver metastases

**DOI:** 10.1016/j.ebiom.2026.106236

**Published:** 2026-03-28

**Authors:** Stavros Makrodimitris, Lissa Wullaert, Daan Hazelaar, Teoman Değer, Ruben G. Boers, Mark A. van de Wiel, Maurice PHM. Jansen, Jaco Kraan, Joachim B. Boers, Wilfred FJ. van IJcken, Corine M. Beaufort, Vanja de Weerd, Stefan Sleijfer, Joost Gribnau, Maarten Vermaas, Eric JT. Belt, Paul D. Gobardhan, Henk MW. Verheul, Dirk J. Grűnhagen, John WM. Martens, Cornelis Verhoef, Saskia M. Wilting

**Affiliations:** a Medical Oncology Erasmus MC Cancer Institute, Erasmus University Medical Center, the Netherlands; b Surgical Oncology and Gastrointestinal Surgery, Erasmus MC Cancer Institute, Erasmus University Medical Center, the Netherlands; c Developmental Biology, Erasmus University Medical Center, the Netherlands; d Epidemiology and Data Science, Amsterdam Public Health, Amsterdam University Medical Center, the Netherlands; e Erasmus Center for Biomics, Erasmus University Medical Center, the Netherlands; f Department of Surgical Oncology and Gastrointestinal Surgery, IJsselland Hospital, the Netherlands; g Department of Surgical Oncology and Gastrointestinal Surgery, Albert Schweitzer Hospital, the Netherlands; h Department of Surgical Oncology and Gastrointestinal Surgery, Amphia Hospital, the Netherlands

**Keywords:** Colorectal liver metastasis, Liquid biopsy, DNA methylation, Copy number variation, Prognostic biomarkers

## Abstract

**Background:**

Half of the patients with colorectal liver metastases (CRLM) that undergo local treatment with curative intent experience recurrence within one year. New biomarkers are needed to stratify patients before and/or after surgery to minimise both over- and under-treatment with peri-operative chemotherapy.

**Methods:**

We profiled 120 patients with CRLM not treated with (neo-)adjuvant chemotherapy using a combined assay for genome-wide cell-free DNA methylation and copy number profiling both pre-operatively and three weeks after local treatment of CRLMs. These data were used to estimate the proportion of circulating tumour DNA (ctDNA) in these patients using data from healthy controls and CRLM tissues for reference. The prognostic value of pre-operative and post-operative ctDNA load was assessed on Recurrence-Free Survival (RFS) and Overall Survival (OS) using Cox proportional hazards models.

**Findings:**

The ctDNA estimates based on both DNA methylation and copy numbers were significantly correlated with mutation-based ctDNA fractions and captured tumour-derived information, such as tumour size. The continuous ctDNA amount estimated using methylation was an independent, pre-operative prognostic marker for both RFS (HR = 1.20, 95% CI = [1.03, 1.39], p-value = 0.019) and OS (HR = 1.31, 95% CI = [1.10, 1.55], p-value = 0.002) after accounting for age, sex, Fong risk score, primary tumour location and metastasis timing. Elevated ctDNA levels post-operatively were significantly associated with shorter RFS (p-value = 0.03), but not OS (p-value = 0.16).

**Interpretation:**

This study demonstrated the prognostic value of pre-operative and post-operative ctDNA in a homogeneous, chemotherapy-naïve cohort of patients with CRLM as well as its potential to guide decisions on administering peri-operative chemotherapy.

**Funding:**

Dutch Cancer Society, Dutch Digestive Health Fund.

**Research in context**

**Evidence before this study**

After primary colorectal cancer treatment, many patients experience metastases confined to the liver. These patients with colorectal liver metastases (CRLM) can still be treated with curative intent. However, around half of these patients exhibit disease recurrence within one year. Randomised trials have shown that (neo-)adjuvant chemotherapy increases Recurrence-Free Survival (RFS), but not Overall Survival (OS). As a result, and in the absence of clinically validated biomarkers, current local guidelines recommend peri-operative chemotherapy for either all patients or for none. Circulating tumour DNA (ctDNA) is a promising, patient-friendly biomarker with an increasing number of studies and trials being carried out in several cancer types.

We queried PubMed for peer-reviewed articles including the terms “colorectal liver metastases” and “circulating tumour DNA”, from database inception until March 4, 2026 without any language restrictions and identified 26 relevant articles after excluding review and opinion articles. A plethora of studies report that post-operative detection of ctDNA is associated with worse RFS and in a subset of those studies also with worse OS, while one study showed that patients with post-operative ctDNA derive more benefit from adjuvant chemotherapy. Evidence is conflicting for the effect of pre-operative ctDNA on RFS and mostly negative for OS, although the vast majority of these studies have been carried out on mixed populations with diverse peri-operative chemotherapy schemes.

Two studies specifically investigated the value of ctDNA during induction chemotherapy and report associations between baseline, on-treatment, and pre-operative (i.e. after induction therapy) ctDNA and early disease recurrence following local therapy as well as OS.

**Added value of this study**

We performed ctDNA analysis on a chemo-naive cohort of 120 patients with CRLM initially eligible for curative treatment using a tumour-agnostic assay. In this homogeneous cohort, we observed a significant association between the baseline (untreated) ctDNA levels and both RFS and OS. This remained true after correcting for post-operative ctDNA levels.

**Implications of all the available evidence**

ctDNA has the potential to guide treatment decisions for patients with CRLM during the whole disease trajectory, from diagnosis until after curative treatment. In addition to detecting post-operative residual disease, it enables upfront identification of a group of patients with poor prognosis at baseline, who might derive benefit from neo-adjuvant treatment, ultimately decreasing both over- and under-treatment.

## Introduction

Colorectal cancer (CRC) is a common and deadly disease, ranking third in world-wide incidence and second in number of deaths among all cancer types.^1^ At the time of initial diagnosis, approximately 20% of patients with CRC will have colorectal liver metastases (CRLM), and an additional 20–30% of patients will develop CRLM during their course of disease.^2^^,^^3^ In about 20% of the cases, CRLMs are treated with curative intent. However, half of these patients face disease recurrence within one year.^4^

Randomised controlled trials showed that peri-operative chemotherapy in patients with resectable CRLM postpones disease recurrence but does not increase overall survival (OS),^5^, ^6^, ^7^ suggesting that only a subset of patients derives survival benefit from additional systemic treatment. On the other hand, such treatments cause substantial short- and long-term toxicity. Various prognostic risk scores based on clinical parameters and/or additional molecular markers (e.g. KRAS/BRAF mutation status) are available and can be applied before surgery,^8^ the most well-known being the score by Fong et al.^9^ However, most clinical guidelines either recommend to administer chemotherapy to all resectable patients or to none, suggesting that current risk models are not used in daily clinical routine, resulting in either over- or undertreatment of patients with CRLM.

Circulating tumour DNA (ctDNA) has recently gained popularity as a tool for up-front prognostication as well as detection of minimal residual disease (MRD) following surgery in patients with cancer.^10^, ^11^, ^12^ ctDNA can be detected in various ways: mutations in (colorectal) cancer-specific genes (using targeted sequencing panels), patient-specific alterations (tumour-informed), or more universal, tumour-agnostic, cancer-specific alterations such as aneuploidy,^13^ DNA methylation,^14^ and fragmentomics.^15^ In resectable CRLM, previous studies have found significant associations between the detection of post-operative, but not pre-operative, ctDNA and worse outcomes.^16^^,^^17^ First proof of the clinical utility of post-surgical, tumour-informed ctDNA detection in resectable CRLM was suggested in a non-randomised subgroup analysis of the GALAXY-trial from 2024, which showed survival benefit for adjuvant chemotherapy specifically in ctDNA-positive patients post-surgery.^18^ However, it should be noted that the investigated patient populations in all these studies were heterogeneous in terms of peri-operative systemic treatments received.

In the Netherlands, peri-operative chemotherapy is not standard of care in patients with resectable CRLM, enabling the evaluation of the true prognostic potential of ctDNA in a homogeneous, chemo-naive population. This inspired the Dutch MIRACLE study, in which pre- and post-operative samples were collected in 188 chemo-naive patients with initially resectable CRLM from four different hospitals.^19^ Prior work in this cohort using detection of mutations by a CRC-specific panel confirmed that post-operative, but not pre-operative detection of ctDNA was associated with worse Recurrence-Free Survival (RFS).^19^ However, the targeted assay used failed to detect ctDNA in 33% of patients before surgery,^19^ whereas results from the GALAXY trial using a tumour-informed ctDNA assay indicated presence of pre-operative ctDNA in almost all patients.^11^ On the other hand, the percentage of post-operatively ctDNA-positive patients was similar in the two studies (27 and 32%).^18^^,^^19^ Although results with tumour-informed ctDNA detection panels are promising in terms of both sensitivity and specificity,^18^ their patient-specific design also renders them expensive and complicated, with considerable turn-around times.

In summary, evidence for the use of ctDNA in the clinical management of patients with CRLM is building up. This necessitates further development of universally applicable, affordable ctDNA detection assays to facilitate worldwide implementation into daily clinical practice. Here, we evaluate a method for simultaneous, tumour-agnostic chromosomal and methylation profiling of cfDNA^20^^,^^21^ to detect and quantify ctDNA in pre- and post-surgical samples of the MIRACLE cohort.

## Methods

### Patients and data

**Fig. 1:**
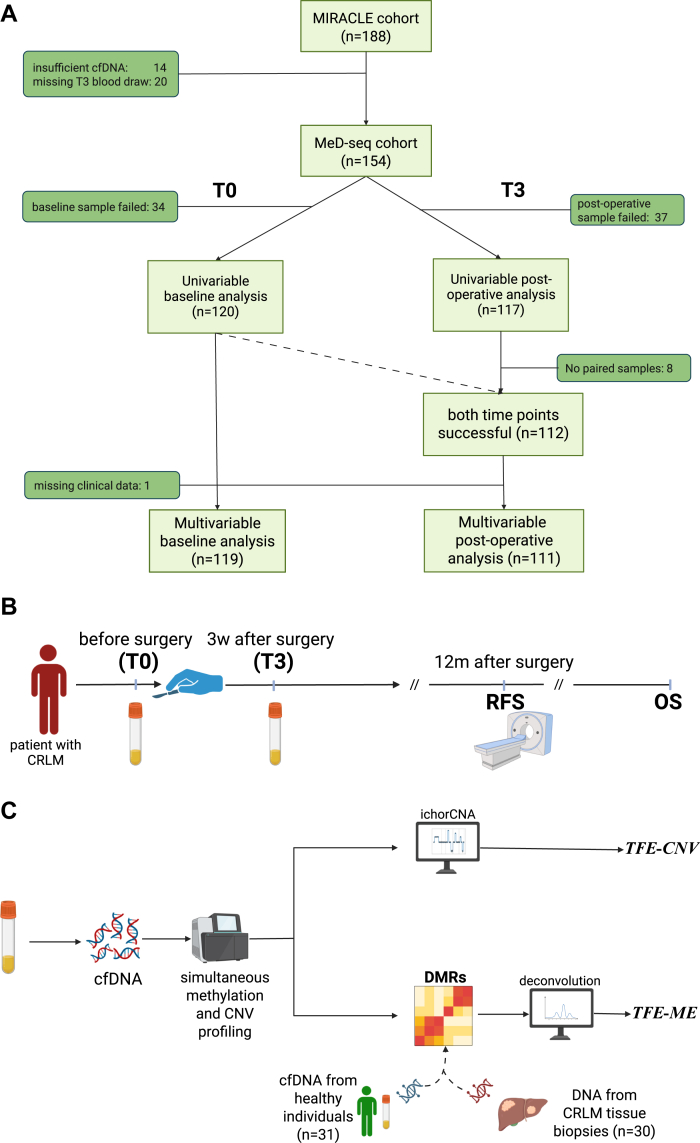
(**A**) Overview of patient samples. (**B**) Timeline of clinical sample collection and follow-up. (CRLM: colorectal liver metastases, RFS: recurrence-free survival, OS: overall survival). Created in BioRender. Oncology, M. (2025). (**C**) Sketch of the approach for tumour fraction estimation using DNA methylation (*TFE-ME*) and copy number variants (*TFE-CNV*). (cfDNA: cell-free DNA, DMRs: differentially methylated regions). Created in BioRender. Oncology, M. (2025).

We included 154 consenting patients with CRLM who were eligible for curative treatment and enrolled in the MIRACLE study,^19^ a prospective, observational biomarker study with four participating Dutch hospitals (Fig. 1A) between October 2015 and December 2021. Patients with CRC diagnosed with resectable, isolated CRLM were eligible to join the study. Patients with extrahepatic disease, liver-first treatments, unresectable CRLMs, or who had received any peri-operative chemotherapy were excluded. Recurrence was diagnosed using radiographic imaging in combination with CEA measurements, according to the Dutch guidelines. For each patient, we collected a pre-operative blood sample less than 24 h before the surgery (T0) and a post-operative sample 3 weeks after surgery (T3, Fig. 1B).

We also included pre-operative blood samples from 12 consenting patients excluded from the MIRACLE cohort due to unresectable disease at time of surgery, administration of adjuvant chemotherapy, or liver-first resections as positive control samples. To compare the cfDNA profiles of our patients to those of healthy individuals, we obtained plasma from 34 volunteers. We additionally used 30 fresh-frozen tissue samples of CRLMs obtained during surgery to define the tumour-specific signal.

### Ethics

The study protocol was approved by the institutional ethics committee at the initiating centre (Erasmus University Medical Center) and at all participating sites (Amphia hospital, IJsselland hospital and Albert Schweitzer hospital) under registration number NL53086.078.15. All participants provided written informed consent in accordance with the principles of the Declaration of Helsinki.

### DNA isolation and methylation profiling

We collected blood in CellSave (Menarini) tubes and processed them to plasma within 96 h by 2 sequential centrifugation steps as described before.^22^ cfDNA was isolated from plasma using the QIAmp Circulating Nucleic Acid kit (Qiagen). For all samples, 10 ng cfDNA was used for methylation profiling using the MeD-seq assay (Fig. 1C).^20^^,^^23^ In short, we digested cfDNA fragments with the methylation-dependent restriction enzyme LpnPI, which creates 32bp-long fragments with a methylated CpG site at their center. Adaptors were ligated followed by a size selection using Pippin (Sage Science) to remove undigested fragments. The resulting libraries were sequenced on either the NextSeq or Hi-seq platform (Illumina) generating approximately 20 M reads per sample. The influence of pre-analytic conditions on MeD-seq cfDNA methylation profiles was evaluated by Deger et al.^20^ Methylation profiling of genomic DNA from CRLM tissues was performed in a similar fashion, as previously described.^23^

LpnPI recognises a methylated CpG at particular sequence contexts (CCG, CCGG, CGG, and GCGC) which corresponds to 16,596,934 locations in the human genome (hg38).^23^ Sequencing reads that had one of the four LpnPI recognition sites 16 bp from either end of the fragment (CpG reads) were mapped to the reference genome. In all our samples, at least 80% (median 98.2%, IQR [97.8%–98.8%]) of the CpG reads were mapped to one of the aforementioned locations. The non-CpG reads were ignored for methylation profiling.

We removed samples for which we suspected that LpnPI digestion was suboptimal or unsuccessful, which we defined as samples consisting of fewer than 20% CpG reads or having fewer than 3,000,000 CpG reads mapped to the human genome.

We then aggregated the methylation levels of nearby CpG sites. This alleviates the noise in the data caused by data sparsity (especially significant in cfDNA analysis) and the “competition” of nearby CpG sites to bind to a given LpnPI molecule. More specifically, we divided the genome into 27,923 regions, each corresponding to a CpG island,^24^ and, per sample, summed the number of reads of all the LpnPI target sites within each region to obtain the total read count for that region. To eliminate sex-specific effects, we only used the 26,840 regions located on the 22 autosomal chromosomes in further analyses.

The per-CpG island read counts are in general over-dispersed, so we chose to model them using negative binomial distributions^21^ and used the edgeR package v3.36^25^ to find differentially methylated regions (DMRs).

To link DMRs to tissue-specific methylation profiles, we used 205 methylation profiles^14^ and converted the genome-wide methylation beta values into a profile of CpG islands by calculating—within each sample—the mean beta value of all CpG sites in each CpG island. Given a list of differentially methylated CpG islands with corresponding log fold changes, we calculated the Spearman correlation between these fold changes and the mean beta value of that CpG island in each sample from the atlas. We then averaged all correlations from samples from the same tissue and sorted the tissues based on these mean correlations.

### Tumour fraction estimation based on copy number variations (CNVs) and mutations

We previously demonstrated that, in addition to methylation profiling, the MeD-seq assay can be used for identifying CNVs.^21^ We binned the genome into 1 Mbp intervals and counted the number of background (non-CpG) reads in each bin. We used LOESS regression to correct for GC content and mappability biases and calculated the log ratio of the corrected read count in each bin to the corresponding median corrected read count in our 31 healthy donors. These log ratios were then used as input for ichorCNA, which returns an estimate of the tumour fraction along with the most likely copy number profile. Following this approach, described in more detail in,^21^ we derived tumour fraction estimates using ichorCNA^13^ (*TFE-CNV*) for all pre- and post-operative patient samples (median coverage for CNV calling 0.1x).

The same patients have been pre-operatively (T0) profiled for hotspot mutations in 14 CRC genes using the Oncomine™ Colon cfDNA Assay (Thermo Fisher Scientific, Waltham, MA, USA) as previously described.^19^ The maximum Variant Allele Frequency (VAF) of all detected mutations was used as a proxy for the tumour fraction in Oncomine™-positive patients. At T3, the presence and quantity of ctDNA in patients with detected Oncomine™ mutations was assessed using digital PCR (dPCR) when available, otherwise using the Oncomine™ panel.^19^ An overview of the numbers of patients with successful methylation and mutation profiles at both time points is provided in Figure S1.

### Tumour fraction estimation using methylation

We assumed that a cfDNA methylation profile is a mixture of two components: healthy cfDNA and ctDNA (Fig. 1C). We first identified DMRs between the two components by comparing 31 cfDNA samples from healthy controls and 30 DNA samples from CRLM tumour tissues using the edgeR package version 3.36.^25^ As healthy cfDNA (mostly consisting of immune cells and progenitors) has vastly different profiles from CRLM tissues, we expected to find a large number of regions with significantly different methylation. To minimise false-positive regions that do not reflect tumour-derived signals, we applied the strict Bonferroni multiple testing correction procedure and selected only regions with a Family-Wise Error Rate (FWER) < 0.05.

To further enrich for tumour-related methylation differences, we used an independent positive control set (n = 10) for which both methylation and mutation data were available. In this dataset, we calculated the correlation between the largest detected Variant Allele Frequency (VAF, median 8%, IQR [2.6%,23.5%]) and the log-TPM normalised^26^ methylation counts and only retained regions with statistically significant correlations (FDR < 0.05) whose sign matched that of the fold-change.

We then calculated the mean and variance of the raw methylation counts in each region in each component and fed these in a negative binomial deconvolution algorithm,^27^ which finds the most likely tumour fraction value (*TFE-ME*, in the range [0.0, 1.0], Supplementary Methods S1).

### Survival analyses

Main outcome of interest was disease recurrence within one year, which was the pre-defined primary end-point of the MIRACLE study.^19^ Therefore, unless otherwise specified, all patients were censored for RFS at 365 days from resection for both Kaplan–Meier and Cox analyses. As a secondary end-point we also evaluated OS.

Multi-variable analyses included age, sex, primary tumour location, KRAS mutational status, whether the CRLM diagnosis was synchronous or metachronous to the diagnosis of the primary tumour, and the risk group according to the Fong score.^9^

The KRAS mutation status of the primary tumour was missing in 75/120 patients, because this information was not standardly collected in all centres at the start of this study. We thus investigated whether using the KRAS mutational status from the Oncomine panel is a good proxy for the primary tumour using the 45 patients for whom we had both measurements. We found that out of 25 KRAS WT patients, all of them were found negative for KRAS mutations by the Oncomine panel (i.e. 100% specificity). Out of 20 KRAS mutant tumours, 17 were also identified as KRAS mutants, leading to a sensitivity of 85% and a total accuracy of 93.3%. The observed large agreement between the assays motivated us to use the liquid biopsy-based KRAS mutational status as a covariate in our multi-variable analyses.

One subject was removed from multi-variable analyses due to missing clinical information (Fig. 1A). The median follow-up time for censored observations was calculated using the method by Schemper and Smith.^28^ For all Cox models, we used the relationship between Schoenfeld residuals and rank-transformed time to test for violations of the proportional hazards assumption.^29^ No significant violations of the assumption were found.

To ease the interpretation of Hazard Ratios (HRs), we scaled the TFEs so that an increase of 1 unit in the scaled variable corresponds to an increase of 10 percentage points to the tumour load (i.e. an increase of 0.1 to the tumour fraction estimate). Age was standardised to zero mean and unit standard deviation.

### Statistics

We tested for differences in the median of a continuous variable between two groups using the Mann–Whitney U test and for overrepresentation of a categorical variable in one of two groups using Fisher's exact test. To test for overrepresentation of genome-wide DMRs (n = 26,840) we used the approximate chi-squared test instead of Fisher's test for computational reasons. The chi-squared test was also used to test for associations of categorical variables with more than two categories. Differences in survival time were assessed using the log rank test.

Where necessary we applied multiple testing correction, controlling for the False Discovery Rate (FDR) using the Benjamini-Hochberg procedure. One exception is the comparison of healthy cfDNA to CRLM tissues, where the stricter Bonferroni correction was applied to correct for the FWER by multiplying all p-values by the number of tests performed.

Given our sample size of 120 patients with 60 RFS events within one year, we performed a power calculation to estimate the minimum hazard ratio that we can identify using the method by Schoenfeld.^30^ In the pre-operative analysis (T0) we split the data into two equal groups, while post-operatively the data were split into two unequal groups (70%–30%). Figure S2 shows that our study had 80% power to detect hazard ratios of at least 2.05 at T0 and at least 2.2 at T3. For OS the number of events was 50 and thus the minimum hazard ratios at 80% power were 2.225 (T0) and 2.375 (T3, Figure S2). The validity of the chosen cut-offs was assessed by bootstrapping and by testing their generalisation across sequencing batches and inclusion centres (Supplementary Methods S2).

### Evaluation and comparison of tumour fraction estimates

We compared the *TFE-ME*, *TFE-CNV*, the Fong score, and our multivariable model using clinical variables and the *TFE-ME* in terms of their ability to model survival outcomes, discriminate between patients with and without an event, and accurately estimate the patients' risks of an event (calibration). We trained Cox models to predict RFS and OS and evaluated them using the concordance index, the ROCAUC and the Integrated Calibration Index (ICI).^31^ Note that higher concordance index and ROC-AUC correspond to a better model, while lower ICI corresponds to a better model, with perfect calibration having an ICI of 0. ROCAUC and ICI were assessed at 12 months for RFS (our study's primary objective) and at 3 years for OS. We employed 10-fold cross-validation stratified by the presence of an event at 1 or 3 years (for RFS and OS respectively) and report the mean and standard error of each metric across the 10 folds.

Furthermore, we employed decision curve analysis^32^ to test whether the predictions of the 3-year OS model including the pre-operative *TFE-ME* could potentially be used to guide clinical decisions regarding administration of neo-adjuvant chemotherapy. Details of this experiment are provided in Supplementary Methods S3.

### Identifying a smaller set of markers for tumour fraction estimation

Starting from the final set of markers that were used for estimating the *TFE-ME*, we tested whether it is possible to obtain comparable tumour fraction estimates using a smaller set of markers, which can be profiled using a more affordable, targeted assay. We used the cfDNA methylation profiles of all 130 patients with CRLM and aimed to build a regression model that predicts the ctDNA load from the log-TPM normalised cfDNA methylation counts in these marker regions. Instead of predicting the *TFE-ME* directly, we first applied the inverse logistic transformation and trained the regressor using the transformed values. This ensures that our model will always make predictions strictly in the range [0, 1] and stabilises the variance of the residuals which is different for values close to 0 or 1 than for values closer to 0.5.

We tried two linear regression models that enforce sparsity and thus perform variable selection, namely least squares with L1 regularisation and least angle regression.^33^ Each model has a single parameter: the amount of L1 regularisation and the maximum number of features with non-zero coefficients, respectively. Model tuning and selection was performed using 3-fold nested cross-validation and the R^2^ as criterion. This allows for unbiased evaluation of the performance of the selected model on unseen data. We then used a single 3-fold cross-validation to select one single best model and parameter that we re-trained on the entire dataset. Finally, the markers that had a non-zero coefficient in that model were selected as potential markers to be used in a targeted assay.

In addition, we tested whether our approach to finding a reduced set of our markers generalises to patients from different hospitals and from different sequencing batches. We categorised patients from the Erasmus MC into one group (Table 1, EMC) and patients from the other three hospitals into a second group (peripheral). We then used one group to find the markers and tested it in the other. We repeated this analysis, but grouped patients based on the sequencing batch, with the first 3 batches (n = 57) constituting one group and the remaining 4 batches (n = 63) a second group.

**Table 1:** Clinical and pathological characteristics by pre-operative *TFE-ME* levels.

Characteristic	*TFE-ME*-high (n = 60)	*TFE-ME*-low (n = 60)	p-value
Median age (years)	66.5	66.0	0.68
Sex			0.12
Male	36	45	
Female	24	15	
Inclusion center			0.69
Albert Schweitzer	4	4	
Amphia	11	12	
Erasmus MC	32	36	
IJsselland	13	8	
DFI from primary to CRLM			0.24
>1 year	45	38	
<1 year	15	22	
Lymph node status primary			1.0
N0	23	23	
N+	36	36	
Number CRLMs			0.14
≥2	36	27	
<2	24	33	
Pre-operative CEA			0.83
High	38	39	
Low	14	16	
Diameter largest CRLM			0.016
≥5 cm	11	2	
<5 cm	49	58	
Resection margin			0.72
R0	55	54	
R1	5	3	
Risk group Fong			0.22
Low	40	47	
High	20	13	
Diagnosis CRLM			1.0
Synchronous	22	21	
Metachronous	38	39	
Location of primary tumour			0.55
Left-sided	28	24	
Rectum	20	19	
Right-sided	12	17	
KRAS status primary tumour			0.04
Wild-type	18	7	
Mutant	8	12	
Unknown	34	41	
BRAF status primary tumour			0.29
Wild-type	25	19	
Mutant	1	0	
Unknown	34	41	

p-values were calculated using the Mann–Whitney U test for continuous variables, Fisher's exact test for binary variables, and the chi-squared test for categorical variables with three or more categories.

### Role of funders

The funders had no role in the study design, data collection, data analyses, interpretation, or writing of the report.

## Results

### Patients

We successfully obtained pre-operative (T0) MeD-seq cfDNA methylation and copy number profiles according to our quality control criteria from 120 patients with CRLM (Fig. 1A, Figure S1), of whom 60 had disease recurrence within one year. Median follow-up for this cohort was 33.9 months for RFS and 60.5 months for OS. The clinical characteristics of this patient cohort are listed in Table 1. This subset of patients is not significantly different from the additional 68 patients included in the original MIRACLE cohort^19^ in both clinical characteristics and outcome, but there is a significant overrepresentation of one inclusion center (Table S1). Similarly, we obtained post-operative (T3) profiles from 117 patients, while for 112 patients, both time points were successfully profiled (Fig. 1A, Figure S1).

### Comparison of healthy cfDNA and CRLM biopsies identifies tumour-specific DMRs

**Fig. 2:**
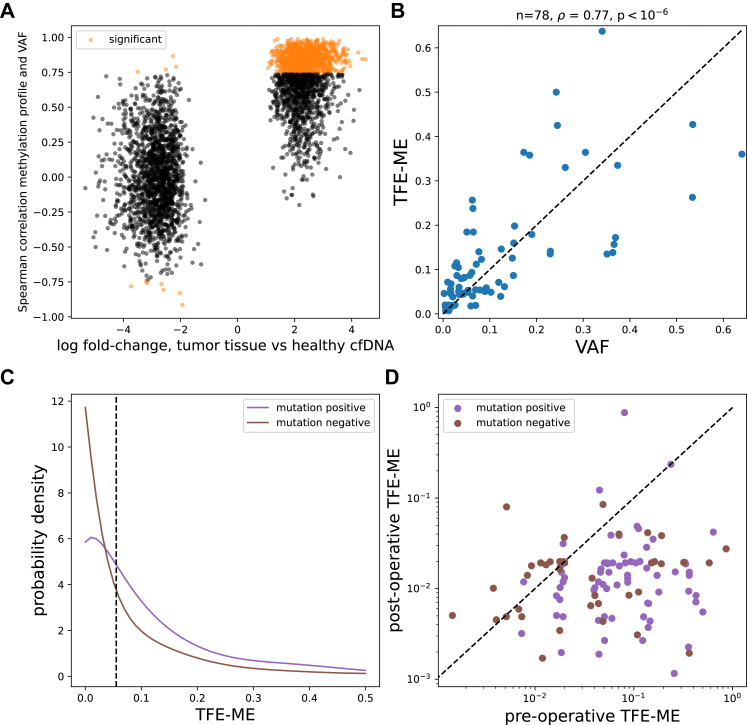
(**A**) Scatter plot in which every point corresponds to a CpG island with its x-co-ordinate corresponding to the fold change of the methylation counts in CRLM tissue vs plasma cfDNA of healthy controls, while the y-co-ordinate shows the Spearman correlation between TPM-normalised methylation values and the VAF in 10 pre-operative cfDNA samples of patients with CRLM. Only CpG islands that are statistically differentially methylated between CRLM tissue and healthy plasma cfDNA after Bonferroni correction are shown (n = 3628). The CpG islands for which the correlation with the VAF was statistically significant (FDR < 0.05 based on the Benjamini-Hochberg method) are shown in orange, while the rest are shown in black. (**B**) Relation of ctDNA load estimates based on mutations (x-axis) and methylation (*TFE-ME*, y-axis) for 78 Oncomine™-positive MIRACLE patients. The black dashed line corresponds to the y = x line. (**C**) Distribution of pre-operative tumour fraction estimates (x-axis) for 78 Oncomine™-positive (purple) and 42 Oncomine™-negative (brown) patients. The y-axis shows the probability density estimates using Beta distribution kernels and bandwidth of 0.04. The median of all 120 TFE*-ME* values is shown as a vertical dashed line. (**D**) The *TFE-ME* of 112 patients with CRLM at T0 (x-axis) versus T3 (y-axis) in logarithmic scale. The black dashed line corresponds to the y = x line. Points are coloured according to baseline mutation positivity status (purple: mutation-positive, brown: mutation-negative).

After filtering, we identified 1069 DMRs between healthy cfDNA and CRLM tissues (Fig. 2A), of which 1062 were hypermethylated in CRLM. This set was enriched mostly for regions on chromosomes 8, 13, and 20 and depleted for regions from chromosome 22. A manually curated list of 28 colorectal-cancer specific methylation markers in cfDNA from the literature (Table S2) was also significantly overrepresented (14/28, p-value < 10^−6^, chi-squared test).

Comparisons with published tissue data^14^ showed that the fold-changes of these DMRs correlated most strongly with methylation profiles from the small and large intestine, while they were most anti-correlated with profiles from granulocytes and erythrocyte progenitors (Figure S3).

### Pre-operative tumour fraction estimates accurately represent ctDNA load

We obtained tumour fraction estimates from copy number data (*TFE-CNV*) using our adaptation of ichorCNA^13^^,^^21^ in 120 pre-operative cfDNA samples from patients with CRLM. In 78 patients (Figure S1) who had a hotspot mutation as previously determined using the Oncomine™ colon panel,^19^ the *TFE-CNV* was significantly correlated with the largest VAF (Spearman's ρ = 0.67, p-value < 10^−6^, Figure S4a).

We also used our set of 1069 DMRs and a deconvolution algorithm to obtain methylation-based tumour fraction estimates (*TFE-ME*), which were even more strongly correlated with the VAF (Spearman ρ = 0.77, p-value < 10^−6^, Fig. 2B). The correlation between the *TFE-CNV* and *TFE-ME* was 0.71 (p-value < 10^−6^), but dropped drastically when recalculated using 1069 random regions instead of the DMRs (Figure S4b).

The *TFE-ME* was on average significantly smaller in Oncomine™-negative patients compared to the Oncomine™-positive ones (median 0.03 vs 0.08, p-value < 0.001, Mann–Whitney U test, Fig. 2C). However, several Oncomine™-negative samples had a high *TFE-ME* (Fig. 2C), implying that perhaps these samples have ctDNA but do not harbour any of the hotspot mutations included in the used mutation panel. On the contrary, the CNV-based method failed to detect ctDNA even in 35% of VAF-positive patients (Figure S4a). These apparent “false negatives” of *TFE-CNV* were on samples with significantly lower VAFs according to the Oncomine™ panel (p-value < 10^−6^, Figure S4c).

Patients whose largest lesion had a diameter larger than 5 cm, as well as patients with a mutation on the APC gene, had significantly higher pre-operative ctDNA load (both *TFE-ME* and *TFE-CNV*, Figures S5–S6, Table S3) compared to patients with smaller lesions and patients without APC mutations respectively. The estimates were not significantly associated with age (ρ = 0.03), or any technical factors (FDR > 0.05, Table S3). Furthermore, *TFE-ME* estimates obtained from two different sequencing runs of the same patient samples were highly similar (n = 12, Spearman's ρ = 0.76, p-value = 0.005, Figure S7). The *TFE-CNV* was also associated with TP53 mutational status, pre-operative CEA, and Fong risk group, while the *TFE-ME* was not. Taken together, these results demonstrate that MeD-seq-derived tumour fraction estimates based on either methylation or copy numbers can accurately capture the circulating tumour load in the blood of patients with CRLM.

### Pre-operative ctDNA levels are prognostic of both RFS and OS

Next, we tested the prognostic value of the pre-operative tumour load assessments. Patients for whom ichorCNA detected ctDNA (*TFE-CNV* > 0, n = 65) had a significantly shorter RFS (8 vs 21 months, p-value = 0.02, log-rank test, HR = 1.85, 95% CI = [1.09, 3.15], Figure S8a). *TFE-CNV*-positive patients had a median OS of 52.9 months, while the median OS was not reached during follow-up for the *TFE-CNV*-negative group (p-value = 0.03, log-rank test, HR = 1.87, 95% CI = [1.05, 3.33], Figure S8b). These effects were no longer statistically significant when using multi-variable analysis including well-known pre-operative CRLM recurrence risk factors (Table S4).

**Fig. 3:**
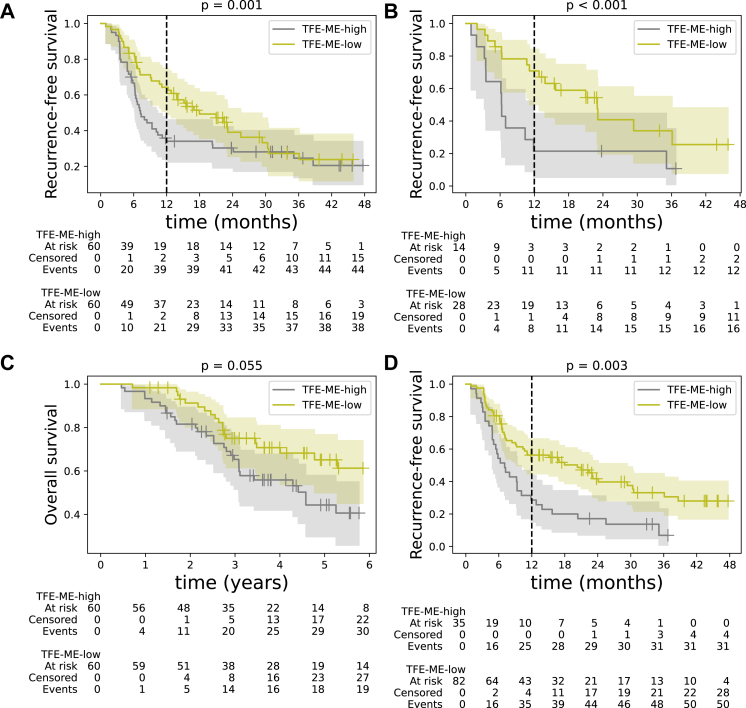
(**A**) Kaplan–Meier curves for RFS for patients (n = 120) that are pre-operatively ctDNA-high (top 50%, grey) and ctDNA-low (bottom 50%, yellow). The 95% confidence intervals of each curve are designated by the shaded areas. (**B**) As in (A), but only for patients who had undetectable ctDNA pre-operatively using the Oncomine™ panel (n = 42). (**C**) As in A) but for overall survival (OS, n = 120). (**D**) Kaplan–Meier curves for RFS for patients (n = 117) that are post-operatively ctDNA-high (top 30%, grey) and ctDNA-low (bottom 70%, yellow). The 95% confidence intervals of each curve are designated by the shaded areas and for RFS curves a dashed vertical line at 12 months signifies that we investigated 1-year RFS. p-values were calculated using the log-rank test.

Focussing on *TFE-ME*, in the absence of a validated cut-off, we split our set of 120 patients into two equally-sized groups (ctDNA-high and ctDNA-low) using the median pre-operative tumour fraction of 5.5%. Kaplan–Meier analysis also showed that the RFS was significantly shorter for ctDNA-high patients compared to ctDNA-low patients (Fig. 3A, median RFS of 7.2 vs 18 months, p-value = 0.001, log rank test, HR = 2.34, 95% CI = [1.38, 3.99]). Both survival curves eventually converge to the mean recurrence rate of this population (about 70–80%) but by this point only a few patients are still at risk in both groups. Bootstrapping showed that using the median *TFE-ME* as a cut-off lies within the 95% confidence interval of the optimal cut-off as determined by maximally selected rank statistics^34^ (Supplementary Methods S2, Figure S9, Table S5) and the median cut-off generalised well across hospitals and sequencing batches (Supplementary Methods S2, Tables S6–S7).

ctDNA was detected in virtually all 42 Oncomine™-negative patients, with 14 (33%) having *TFE-ME* greater than the median of 5.5% (Fig. 2C). These *TFE-ME*-high patients had a significantly shorter RFS than the remaining Oncomine™-negative, *TFE-ME*-low patients (median RFS of 6.3 vs 23 months, p-value < 0.001, log-rank test, HR = 4.17, 95% CI = [1.66, 10.45], Fig. 3B), despite the reduced power due to analysing a smaller set of patients.

An increase of 0.1 to the continuous *TFE-ME* leads to a 21% increase in the risk of recurrence within one year according to a Cox model (HR = 1.21, 95% CI = [1.05, 1.40], p-value = 0.01). This effect remained statistically significant with a similar HR when performing a multi-variable analysis (Table 2).

**Table 2:** Multi-variable analysis for RFS and OS using the continuous pre-operative *TFE-ME* (T0, n = 120, top) and both pre-operative and post-operative *TFE-ME* (T0 and T3, n = 111, bottom).

Variable	RFS			OS		
	HR	95% CI	p-value	HR	95% CI	p-value
Age (standardised)	1.12	[0.83, 1.52]	0.72	1.48	[1.05, 2.09]	0.027
Sex male	0.90	[0.52, 1.56]	0.70	0.67	[0.36, 1.23]	0.19
*TFE-ME* (cont.)	**1.22**	**[1.04, 1.42]**	**0.012**	**1.35**	**[1.13, 1.60]**	**0.0009**
Fong high risk	**2.30**	**[1.32, 4.00]**	**0.003**	**1.83**	**[1.01, 3.33]**	**0.046**
Right-sided tumour (ref. left-sided)	0.71	[0.37, 1.34]	0.29	1.12	[0.55, 2.26]	0.76
Rectal tumour (ref. left-sided)	**0.50**	**[0.26, 0.97]**	**0.04**	1.09	[0.53, 2.24]	0.81
Metachronous metastasis	0.62	[0.34, 1.14]	0.12	0.70	[0.36, 1.35]	0.29
KRAS mutant	1.44	[0.81, 2.56]	0.22	1.69	[0.91, 3.13]	0.097
**T0 and T3**						

Age (standardised)	1.22	[0.89, 1.68]	0.22	**1.61**	**[1.12, 2.33]**	**0.01**
Sex male	0.86	[0.48, 1.53]	0.61	0.77	[0.41, 1.44]	0.41
*TFE-ME*@T0 (cont.)	**1.20**	**[1.02, 1.42]**	**0.024**	**1.36**	**[1.14, 1.62]**	**0.0006**
*TFE-ME*@T3 (cut-off)	2.30	[1.28, 4.11]	0.005	1.43	[0.74, 2.73]	0.29
Fong high risk	**2.79**	**[1.56, 5.00]**	**0.0006**	**2.02**	**[1.10, 3.73]**	**0.023**
Right-sided tumour (ref. left-sided)	0.67	[0.34, 1.29]	0.231	1.06	[0.51, 2.17]	0.88
Rectal tumour (ref. left-sided)	**0.46**	**[0.23, 0.91]**	**0.027**	1.00	[0.48, 2.12]	0.99
Metachronous metastasis	0.59	[0.31, 1.11]	0.10	0.69	[0.35, 1.34]	0.27
KRAS mutant	1.47	[0.80, 2.70]	0.22	1.79	[0.93, 3.47]	0.08

KRAS mutational status was determined by liquid biopsy. p-values < 0.05 are shown in bold.

With respect to OS, the *TFE-ME*-high group had a median survival of 55 months, while the median survival of the *TFE-ME*-low group was not reached during the follow-up period (HR = 1.73, 95% CI = [0.98, 3.05], p-value = 0.055, log-rank test, Fig. 3C). The median cut-off lay again within the 95% confidence interval of the optimal cut-off for OS (Figure S9, Table S5). Using the continuous estimates, we did find a statistically significant effect of a 26% increase in risk for every increase of 0.1 in the *TFE-ME* (HR = 1.26, 95% CI = [1.08, 1.47], p-value = 0.003), which remained significant in a multi-variable analysis (Table 2).

Ten-fold cross-validation showed that the *TFE-ME* outperforms the *TFE-CNV* on both RFS and OS based on the concordance index. It was also more accurate and better calibrated at identifying patients with a 1-year RFS event or a 3-year OS event, although the mean metrics are in some cases within one standard error of each other (Figures S10–S11, Table S8). We further found that the multivariable model with clinical parameters and *TFE-ME* was the best at modelling survival outcomes, achieving the highest concordance index. It was also the best at identifying OS events within 3 years (Figure S11) and performed on par with methylation only at identifying RFS events within one year (Figure S10, Table S8). The calibration of the multi-variable RFS model was less good compared to both *TFE-ME*-only and Fong-only, but it was competitive for OS (Figures S10–S11, Table S8). This might be due to the use of many uninformative features (sided-ness, sex, etc), which hampers calibration. For both tasks, the *TFE-ME* model led to the best calibration.

The potential clinical utility of the above multi-variable OS model (Table 2) in guiding decisions on administering neo-adjuvant chemotherapy (NAC) was investigated by decision curve analysis (Supplementary Methods S3).^32^ Using the model's cross-validated estimates of risk of death within three years from surgery, outperformed treating everyone and treating no-one in terms of net benefit for the entire range of clinically relevant cut-offs,^32^ while the Fong score alone demonstrated a clearly inferior decision curve (Figure S12). Together, these results show that the *TFE-ME* is an independent pre-operative prognostic marker for CRLM with potential clinical utility.

### Detection of post-operative ctDNA is associated with shorter RFS

We applied the same deconvolution algorithm to cfDNA samples collected approximately three weeks after local treatment from the same patient cohort. The *TFE-ME* was reduced post-operatively (mean decrease of 30%, p-value < 10^−6^, Wilcoxon signed rank test, Fig. 2D). Of 117 successful MeD-seq runs, only 6% had a *TFE-ME* larger than the pre-operative median. The *TFE-CNV* was zero in all T3 samples except for one, confirming the limited sensitivity of copy number profiling for minimal residual disease detection. The single *TFE-CNV*-positive patient at T3 tested negative for ctDNA based on mutations.

Due to the significantly lower post-operative *TFE-ME* values, we sought an alternative cut-off when evaluating post-operative prognostic performance. Previous works found the post-operative ctDNA positivity to be 27%^19^ or 32%,^18^ motivating us to define the cut-off so that 30% of our patients are labelled *TFE-ME*-positive, which corresponds to 1.9%. 35% of mutation-positive patients at T3 were also positive for methylation based on this cut-off, while 23% of mutation-negatives were methylation-positive (p-value = 0.38, Fisher's exact test), which might hint that methylation and mutations capture partly different information. Among patients without an Oncomine™ hit at baseline, 27% were ctDNA-positive post-operatively based on methylation, which is comparable to the post-operative detection rate in baseline Oncomine™-positive patients. Finally, the set of post-operatively *TFE-ME*-positive patients was enriched for pre-operatively *TFE-ME*-high patients (p-value = 0.03, Fisher's exact test, Figure S13).

The median RFS for the methylation-positive group (n = 35) was 7 months versus 20.3 months for methylation-negative patients, (p-value = 0.003, log-rank test, Fig. 3D). For 75 patients we could assess both mutation and methylation status at T3. This analysis revealed that patients with a double negative status performed best, whereas a small set of 7 patients (9.6%) that were both mutation-positive and methylation-positive all had recurrent disease within 10.5 months (Figure S14, Table S9).

For multivariable analyses, we used 111 patients with successful MeD-seq runs in both time points and no missing clinical information (Fig. 1A). Patients above the post-operative cut-off of 1.9% had significantly worse 1-year RFS after correcting for clinical factors and the pre-operative *TFE-ME* (HR = 2.41, Table 2). However, this did not translate to a significantly shorter OS (Table 2). The pre-operative ctDNA levels were still a significant prognostic factor for both OS and RFS after including the post-operative measurement (Table 2). This implies that there is complementary prognostic information in the two measurements.

Despite the small range of values of the continuous *TFE-ME* at T3, the log-transformed, continuous post-operative ctDNA levels were also significantly associated with RFS (Table S10), while Table S5 shows that our choice of the 70th percentile lies within the 95% confidence interval of the optimal post-operative cut-off for both 1-year RFS and OS. These results consolidate the prognostic value of *TFE-ME* both pre-operatively and post-operatively.

### Calculation of *TFE-ME* is possible using 43 markers

We then investigated whether we could reduce the number of markers to facilitate the development of a targeted assay. Via a data-driven feature selection procedure, we obtained a model with 43 markers (Table S11). The best model was a least squares regression with L1 penalty, which achieved a mean R^2^ of 0.9 on predicting the *TFE-ME* on unseen data based on nested cross-validation. The selected markers included regulatory regions associated with the silencing of well-known tumour suppressor genes, such as HAND2-AS1,^35^ KANK1^36^ (enhancers), and LIFR (promoter).^37^ The full list of selected regions is provided in Table S11. This result shows that genome-wide methylation profiling of cfDNA might not be necessary for accurate tumour fraction estimation.

By further testing this approach, we observed that it generalises well to unseen samples from different sequencing batches and hospitals (ρ > 0.84, Table S12), while selecting a similar number of markers in each iteration (24–67, Table S12). Moreover, when using the predicted *TFE-ME* from our model on the test data for predicting OS, we found hazard ratios close to 1.31 which we obtained on the entire dataset using the deconvolution-based estimates (1.24–1.66, Table S12).

We then tested whether this regression model using 43 markers, which was built on only pre-operative samples, generalises to the post-operative time point. We found that the Spearman correlation between the *TFE-ME* by deconvolution and using the 43 markers was 0.642 (p-value < 0.001), while the 70-th percentile cut-off was also conserved (0.018% ctDNA using the 43 markers vs 0.019% from the deconvolution using all data). Out of the 117 patients tested, the 1-year recurrence rate in the group with the top-30% *TFE-ME* based on the 43 markers was 66%, vs 45% in the bottom 70% group, signifying a statistical overrepresentation of quicker recurrence in the *TFE-ME*-positive group (p-value = 0.04, Fisher's exact test).

## Discussion

This paper describes a new genome-wide tumour-agnostic method to quantify the circulating tumour load in chemo-naive patients with CRLM using methylation and CNVs. Applying this method in 120 patients, we showed that pre-operative ctDNA levels are prognostic of both 1-year RFS and OS. Methylation performed better than CNVs, which displayed lower sensitivity at the sequencing depth of 0.1x and high correlation with the Fong score, while methylation was a more accurate, independent marker according to multi-variable analyses.

Previous work using mutations in the same cohort found no association between the detection of pre-operative ctDNA–as determined by a CRC-specific panel detecting mutations - and recurrence-free survival, but did not investigate the ctDNA amount.^19^ A meta-analysis of three independent studies reached the same conclusion in patient populations undergoing diverse peri-operative treatment schemes.^38^ Another small study with 34 patients found an association between RFS and ctDNA dynamics during NAC, but not the baseline levels.^39^ A prospective study using a targeted methylation assay found that baseline ctDNA-positive patients had significantly shorter RFS, but not OS.^40^ However, that study did not investigate ctDNA levels and used a mixed population with two thirds of the patients receiving NAC,^40^ so their findings may partly reflect the response to NAC and not the pure effect of baseline ctDNA. Our finding about OS is supported by a large study which found that patients with more than 10% ctDNA at baseline have significantly worse OS,^10^ although this study was carried out on a different population of metastatic CRC patients in terms of stage and treatment.

Elevated post-operative ctDNA was associated with worse recurrence-free survival, confirming previous studies that used mutations^18^^,^^19^ or methylation.^17^ We further observed that post-operative *TFE-ME* carries added value with respect to mutations, with patients who were positive for both assays all having recurrent disease within one year from surgery, although the small group sizes mean that this observation requires additional validation. We did not find evidence that the post-operatively ctDNA-high group also experienced shorter OS.

Most importantly, the pre-operative ctDNA levels are a strong, independent prognostic factor, even after including the post-operative ctDNA levels. Since compliance to chemotherapy is known to decrease in case of post-surgical complications,^18^ NAC may represent the preferable strategy. Survival and decision curve analyses confirmed that combining baseline ctDNA levels with clinical parameters can potentially guide treatment decisions and reduce over- or undertreatment depending on current local guidelines. A future trial could thus test the OS benefit of NAC in high-risk patients as determined by ctDNA and clinical risk factors. Other studies suggest that ctDNA can potentially be useful for monitoring patients during NAC as well,^39^^,^^41^^,^^42^ which might identify a sub-group of patients who stay ctDNA-high and may represent candidates for novel treatment options like transplantation potentially combined with chemotherapy.^43^ Although ctDNA evaluations in the context of liver transplantation in patients with CRLM are limited, first studies suggest that following transplantation, part of the patients revert from ctDNA+ to ctDNA−.^43^^,^^44^ On the other hand, high pre-operative ctDNA levels are associated with poor prognosis and as such may guide selection of candidates for transplantation.^45^ Separate analyses in the MIRACLE indicate high pre-operative ctDNA levels are associated with higher chances of multi-organ recurrence, suggesting these patients cannot be salvaged by local treatment alone.^46^

Using MeD-seq and our algorithm, we were able to detect ctDNA in virtually all patients versus 66% with the Oncomine™ assay which only assesses common CRC variants in 14 genes.^19^ This implies that most chemo-naive patients with CRLM have ctDNA which could have also been detected by mutations using a larger or a personalised panel. In the GALAXY study, the pre-operative ctDNA detection rate was 98% -albeit in a slightly different setting.^11^

A downside of our approach is the assay's failure rate (∼1 in 5), which is too large for direct clinical implementation. However, this rate is inflated due to technical issues related to the flow cell used in one sequencing batch. Previous experiences with this assay and cfDNA demonstrate a lower failure rate of around 8%. Moreover, we showed that we can obtain highly concordant tumour fraction estimates using only 43 markers out of the 1069 initially identified. Building a targeted assay using these markers might increase the reliability and decrease the cost of the current assay.

Another limitation is the lack of established cut-offs for the *TFE-ME*. We defined the cut-offs so that we obtain two equal groups pre-operatively and using prior knowledge from the literature^18^^,^^19^ about the fraction of ctDNA-positive patients post-operatively. The fact that the continuous tumour fraction levels, which do not suffer from information loss due to dichotomisation,^47^ were also significantly associated with the outcomes of interest strongly supports the conclusions drawn using the cut-offs. Nonetheless, external validation in independent cohorts is required before the method can enter clinical practice, also including cohorts that receive peri-operative chemotherapy.

Finally, non-tumour-related cell death caused by surgical trauma significantly dilutes the tumour-derived signal for up to four weeks after surgery.^48^ Our sampling at 3 weeks might thus have hampered our ability to detect significant differences in OS between MRD-positive and MRD-negative patients. For example, Kataoka et al. did find such an effect, having collected the post-operative samples up to 10 weeks after surgery, but they also used a more sensitive, tumour-informed assay.^18^ Thus, the accuracy of tumour-agnostic MRD detection could be improved by delaying the post-operative blood draw. Future studies should optimise the sampling timing to balance accurate MRD detection with timely initiation of adjuvant chemotherapy, as has been done for primary CRC.^49^

In conclusion, the presented tumour-agnostic method of detecting and quantifying circulating tumour DNA provides a minimally invasive, independent prognostic biomarker for both disease recurrence and overall survival. Upon validation in independent cohorts, randomised trials should confirm the clinical utility of ctDNA-guided treatment decisions in the management of resectable CRLM patients. Our results constitute a step closer to personalising treatment strategies for patients with CRLM.

## Contributors

SM and SMW have accessed and verified all underlying data. All authors read and approved the final version of the manuscript. Detailed author contributions are described below:

Conceptualisation: SMW, CV, JWMM, SS, SM.

Methodology: SM, MvdW, DH, JBB, SMW.

Software: SM, MvdW, DH, JBB.

Validation: SM, SMW.

Formal analysis: SM.

Investigation: TD, CMB, VdW, SM, LW, DH, MPHMJ, JK.

Resources: WFJvIJ, RGB, SS, JG, MV, EJTB, PDG, HMWV, DJG, JWMM, CV.

Data Curation: LW, SM.

Writing—Original Draft: SM, SMW.

Writing—Review & Editing: all authors.

Visualisation: SM.

Supervision: SMW, CV.

Project administration: SMW, LW, JK.

Funding acquisition: CV, SMW, SS.

## Data sharing statement

Raw sequencing data are available via the Sequencing Read Archive (SRA, BioProject ID: PRJNA1403605). Processed sequencing data and the open-source code to reproduce the statistical analyses are available at https://github.com/stamakro/miracle-medseq. Clinical data, including de-identified individual participant data can be made available to researchers upon reasonable request. Data enquiries should be directed to the corresponding author.

## Declaration of interests

RGB and JB are founders and shareholders of Methylomics B.V., a company that uses MeD-seq for cancer diagnostics. WFJvIJ is shareholder of Methylomics B.V. and JG has received consulting fees from Methylomics B.V. The remaining authors declare no conflicts of interest.
