# Supplementary material for: Prognostic value of peri-operative circulating tumour DNA levels estimated by cell-free DNA methylation in patients with resectable colorectal liver metastases
Source: eBioMedicine. 2026 Mar 28;126:106236. doi: 10.1016/j.ebiom.2026.106236 (PMC13054272; doi:10.1016/j.ebiom.2026.106236)
Supplement: Supplementary Materials [file mmc1.pdf]

Prognostic value of peri-operative circulating tumour DNA levels estimated by cell-free DNA methylation in patients with resectable colorectal liver metastases

Supplementary Material

Stavros Makrodimitris[1], Lissa Wullaert†[2], Daan Hazelaar†[1], Teoman Değer[1], Ruben G Boers[3], Mark A van de Wiel[4], Maurice PHM Jansen[1], Jaco Kraan[1], Joachim B Boers[3], Wilfred FJ van IJcken[5], Corine M Beaufort[1], Vanja de Weerd[1], Stefan Sleijfer[1], Joost Gribnau[3], Maarten Vermaas[6], Eric JT Belt[7], Paul D Gobardhan[8], Henk MW Verheul[1], Dirk J Grünhagen[2], John WM Martens[1], Cornelis Verhoef‡[2], and Saskia M Wilting‡[1]

[1]Medical Oncology, Erasmus MC Cancer Institute, Erasmus University Medical Center
[2]Surgical Oncology and Gastrointestinal Surgery, Erasmus MC Cancer Institute, Erasmus University Medical Center
[3]Developmental Biology, Erasmus University Medical Center
[4]Epidemiology and Data Science, Amsterdam University Medical Center
[5]Erasmus Center for Biomics, Erasmus University Medical Center
[6]Department of Surgical Oncology and Gastrointestinal Surgery, IJsselland Hospital
[7]Department of Surgical Oncology and Gastrointestinal Surgery, Albert Schweitzer Hospital
[8]Department of Surgical Oncology and Gastrointestinal Surgery, Amphia Hospital

Contents

Supplementary Methods **3**
1. Mathematical description of the deconvolution method 3
2. Cut-off validity and optimization 3
3. Decision curve analysis 4

Supplementary Figures **6**

Supplementary Tables **19**

References **28**

Supplementary Methods

1. Mathematical description of the deconvolution method

The algorithm finds the tumor fraction value (in the range [0.0, 1.0]) that maximizes the log-likelihood of the observed cfDNA methylation profile conditioned on the estimated means and variances (expressed in terms of the mean and overdispersion parameter) of the two components. We employed shrinkage to regularize the overdispersion estimates as in [8]. Below we summarize the mathematical description of the algorithm.

First, we model measured methylation M_{ij} for individual i and methylation marker j as a convolution:

$$M_{ij} = \pi_i T_{ij} + (1 - \pi_i) B_{ij},$$

with π_i the tumor fraction (TFE-ME), T_{ij} the tumor-specific methylation signal and B_{ij} the background, non-tumor methylation signal. Next, we assume

$$T_{ij} \sim \mathrm{NB}(\boldsymbol{\theta}_j^T) \text{ and } B_{ij} \sim \mathrm{NB}(\boldsymbol{\theta}_j^B),$$

with NB denoting the negative binomial distribution and $\boldsymbol{\theta}_j^T = (\mu_j^T, \phi_j^T), \boldsymbol{\theta}_j^B = (\mu_j^B, \phi_j^B)$, the known mean and overdispersion parameters. To estimate $\boldsymbol{\pi} = (\pi_i)_{i=1}^n$, we maximize the pseudo-log-likelihood:

$$\ell_{\Theta^T,\Theta^B}(M; \boldsymbol{\pi}) = \sum_{i=1}^{n} \sum_{j=1}^{p} \log\bigl(P_{\pi_i,\boldsymbol{\theta}_j^T,\boldsymbol{\theta}_j^B}(M_{ij} = m_{ij})\bigr),$$

with M: the entire $n \times p$ data matrix for the p selected methylation markers and Θ^T, Θ^B containing all known distribution parameters $\boldsymbol{\theta}_j^T, \boldsymbol{\theta}_j^B$. Here, $\ell_{\Theta^T,\Theta^B}(M; \boldsymbol{\pi})$ is a *pseudo*-log-likelihood as it assumes independence between the markers. Such pseudo-log-likelihoods are routinely used to estimate parameters, in particular when the dependency structure is unknown or hard to estimate.

As M_{ij} is a weighted convolution of two independent NB random variables, $P_{\pi_i,\boldsymbol{\theta}_j^T,\boldsymbol{\theta}_j^B}(M_{ij} = m_{ij})$ is efficiently evaluated by using probability generating functions (PGFs), as detailed in [1], Suppl Note 1. For very large values of m_{ij} such a calculation may still be time-consuming. A fast and accurate approximation is obtained by binning the values $0, \ldots, m_{ij}$ in 100 bins, and then applying the PGFs for the bins, using the cumulative component-specific NB probabilities per bin. This allows fast computation of $P_{\pi_i,\boldsymbol{\theta}_j^T,\boldsymbol{\theta}_j^B}(M_{ij} = m_{ij})$ for every proposal of π_i, and hence fast evaluation of $\ell_{\Theta^T,\Theta^B}(M; \boldsymbol{\pi})$. Finally, we use the `R`-package `Rsolnp` to perform constrained maximization of $\ell_{\Theta^T,\Theta^B}(M; \boldsymbol{\pi})$ with respect to $\boldsymbol{\pi}$, constraining $0 \leq \pi_i \leq 1$ for all $i = 1, \ldots n$.

2. Cut-off validity and optimization

At baseline, we chose the median of all T0 TFE-ME values in order to have two equal groups, which we then labeled TFE-ME-high and TFE-ME-low. Post-

operatively, the cut-off was chosen based on two previous studies on the same patient population which found that about 30% of patients were post-operatively ctDNA-positive. To test the validity of our cut-offs for the TFE-ME, we used maximally selected rank statistics to identify the optimal cut-off [5]. In the absence of external validation, optimizing the cut-off on a single dataset is susceptible to overfitting and does not guarantee optimality on new patients. We thus used bootstrapping to assess the variability of the optimal cut-off when repeating the experiment. We sampled with replacement from our original dataset, re-ran the cut-off optimization procedure on the bootstrapped data, and recorded the optimal cut-off in that case. We repeated that process 1,000 times (separately for OS and RFS, and separately for T0 and T3) and used the bootstraps to construct 95% confidence intervals for the optimal cut-offs. Table S5 and Figure S9 show that our chosen thresholds lie within the 95% confidence interval of the optimal threshold for both time points and outcomes (RFS and OS).

Furthermore, we simulated external validation by testing whether using the median cut-off pre-operatively generalizes to patients from different hospitals and from different sequencing batches. On the per hospital analysis, 68 of our patients come from one center (Erasmus MC), while the remaining 52 come from three others (Table 1, main document). To avoid having very small groups, we categorized patients from Erasmus MC into one group (EMC) and patients from the other three hospitals into a second group (peripheral). We then used one group to define the cut-off and tested it in the other. We repeated this analysis, but grouped patients based on the sequencing batch, with G1 corresponding to the first 3 batches (n=57) and G2 the remaining 4 batches (n=63). The results (Tables S6 and S7)are similar to our findings on the entire dataset. Using part of the dataset to establish the pre-operative cut-off using the median leads to significant differences in 1-year RFS between TFE-ME-high and -low patients in the held-out part. This is true both across batches and across hospitals. On the other hand, splitting the entire dataset based on the median cut-off did not lead to significantly different OS durations and thus our results on OS in this sub-analysis are mixed. This experiment further supports the validity of the median cut-off.

3. Decision curve analysis

We performed net benefit analysis as described in [9], to test whether a ctDNA-based model could guide decisions on administering neo-adjuvant chemotherapy in patients with resectable CRLM. As currently there is no evidence of overall, population-wide OS benefit from this treatment, we seek for a subset of high-risk patients who might benefit from it and compare this strategy to treating everyone and treating no-one in terms of net benefit [9]. Net benefit is defined as the number of treated patients who would benefit from the treatment minus the treated patients who would not benefit, weighed by a coefficient that depends on the efficacy of the treatment and the severity of the outcome [10]. As both of these parameters are unknown and hard to estimate, decision curves study

net benefit on a wide range of cut-off probabilities. Only if the net benefit curve of a model or diagnostic test is superior or equal to the curve of both 'treat all' and 'treat none' strategies for the entire range of cut-offs that are relevant for clinical decision-making, can we conclude that the model/test has potential to guide decisions [10].

Here, we evaluated the multi-variable Cox model of OS (Table 2, main text) using the continuous pre-operative *TFE-ME*, Fong risk group, age, sex, tumor sided-ness, whether the metastasis was synchronous or metachronous, and KRAS status. As the median survival time in this population is longer than 5 years, we defined death within 3 years from surgery as an outcome to potentially guide the treatment decision.

To avoid overfitting, we estimated risk of death using 10-fold cross-validation. In each fold, 90% of the patients were used to train the Cox model and the model then estimated the probability of having an OS event within 3 years for the remaining 10%. The internally-validated risk estimates from all folds were subsequently combined to calculate a single net benefit curve for a wide range of clinically relevant thresholds (Figure S12).

Supplementary Figures

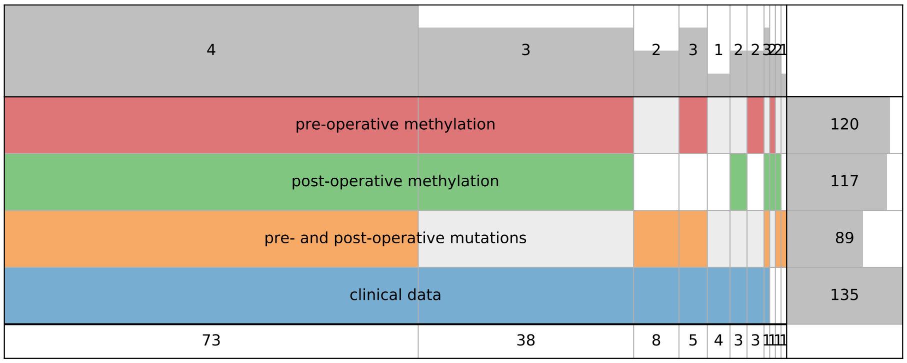

Figure S1: Overview of which sample types ($y-$axis) are available for which patients ($x-$axis). The presence of clinical data is indicated in blue, mutation data in orange, pre-operative methylation in red and post-operative methylation in green. If a sample is not available or failed for a patient, it is shown as white and sets of samples that have the same measurements available are grouped together between gray vertical lines. The gray bars on top of each group indicate how many measurement types are available for that group, while the gray bars on the right denote how many patient samples are in total available for each measurement.

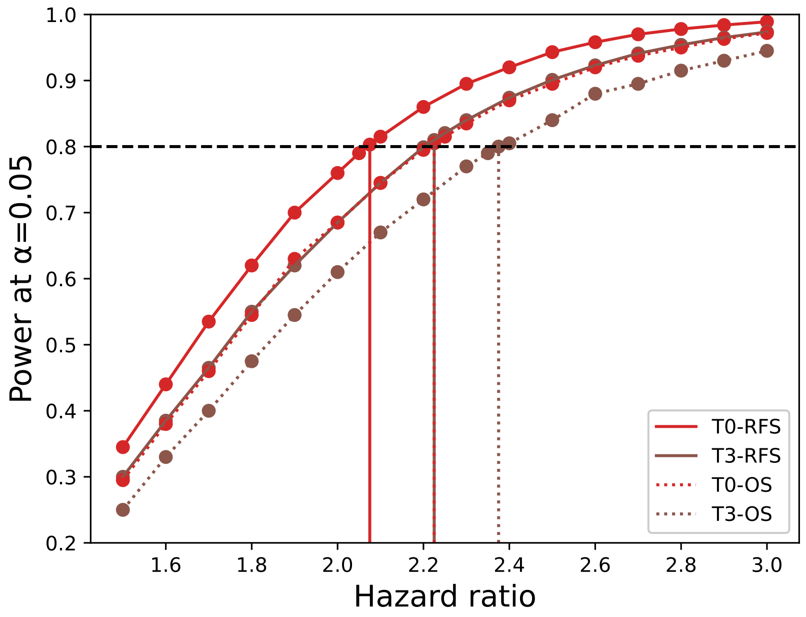

Figure S2: Power (probability of correctly rejecting the null hypothesis, $y-$axis) of our study to detect a particular hazard ratio ($x-$axis). The results for RFS and OS are shown with a solid and a dotted line respectively, while red curves denote the power at T0 (pre-operatively) and brown curves the power at T3 (post-operatively).

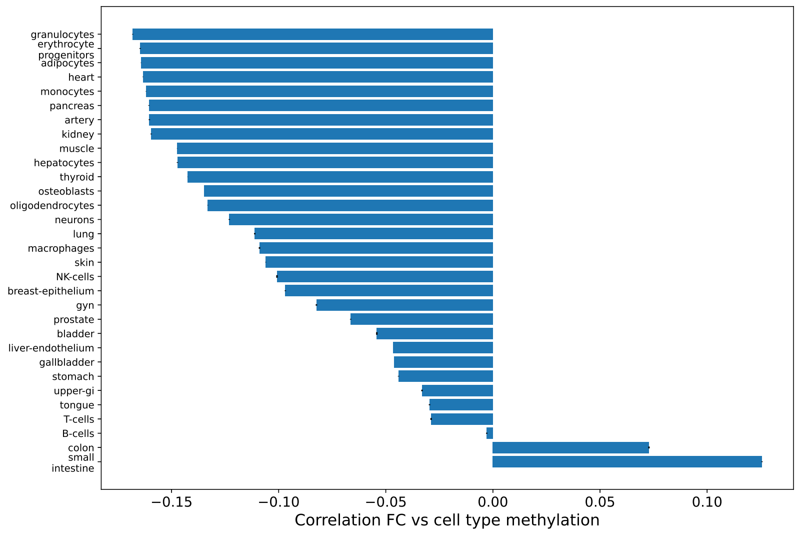

Figure S3: Correlations (x-axis) between methylation beta values in a particular tissue (y-axis) and the log fold-changes between CRLM tissues and healthy cfDNA for 1,069 DMRs. The correlations were calculated per sample and the bars show the mean across all samples from the same tissue/cell type, while error bars denote the standard deviations across samples.

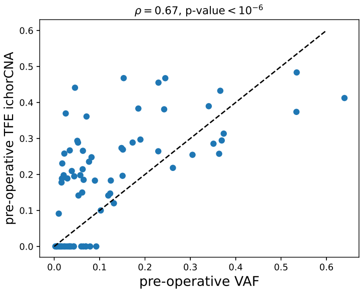

(a) Relationship between the pre-operative variant allele frequency (x-axis) and the *TFE-CNV* (y-axis) for 78 Oncomine positive patients. The $y = x$ line is denoted by a dashed black line.

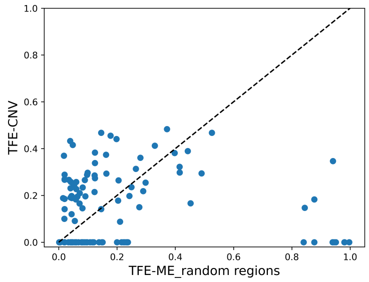

(b) Relationship between the pre-operative *TFE-CNV* (y-axis) and the *TFE-ME* (x-axis) calculated using 1,069 random regions instead of the DMRs (Spearman's ρ=0.15, p-value=0.10).

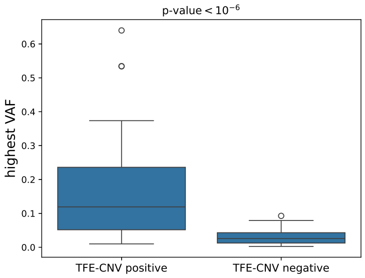

(c) Distribution of pre-operative Variant Allele Frequencies (VAF, $y-$axis) for 78 VAF-positive patients with and without detectable ctDNA using the *TFE-CNV*.

Figure S4

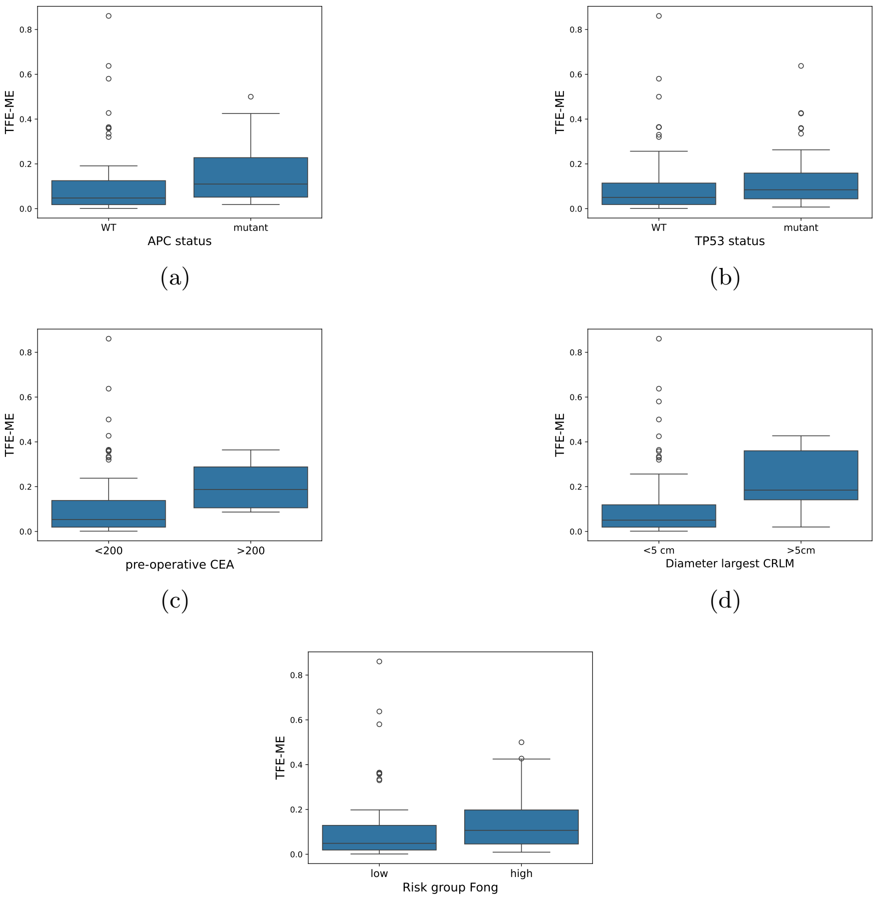

(a) (b) (c) (d) (e)

Figure S5: Distribution of *TFE-ME* (y-axis) according to a) APC mutational status (x-axis), b) TP53 mutational status, pre-operative CEA, d) diameter of largest CRLM, and e) Fong risk group. f) The percentage of replacement growth pattern ('$x-$axis) related to the *TFE-ME* ($y-$axis). The 2-dimensional density is also shown with darker blue corresponding to higher density of points.

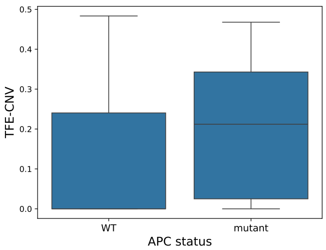

(a)

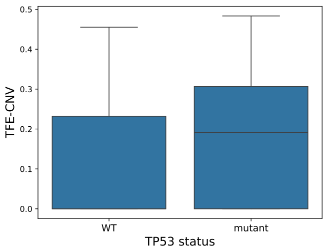

(b)

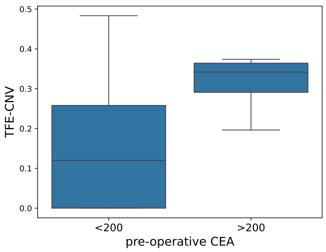

(c)

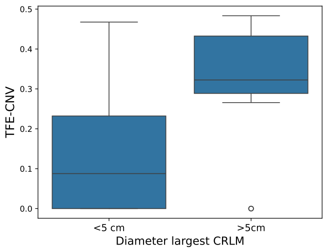

(d)

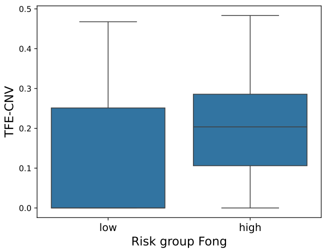

(e)

Figure S6: Distribution of *TFE-CNV* (y-axis) according to a) APC mutational status (x-axis), b) TP53 mutational status, pre-operative CEA, d) diameter of largest CRLM, and e) Fong risk group. f) The percentage of replacement growth pattern ($x-$axis) related to the *TFE-CNV* ($y-$axis). The 2-dimensional density is also shown with darker blue corresponding to higher density of points.

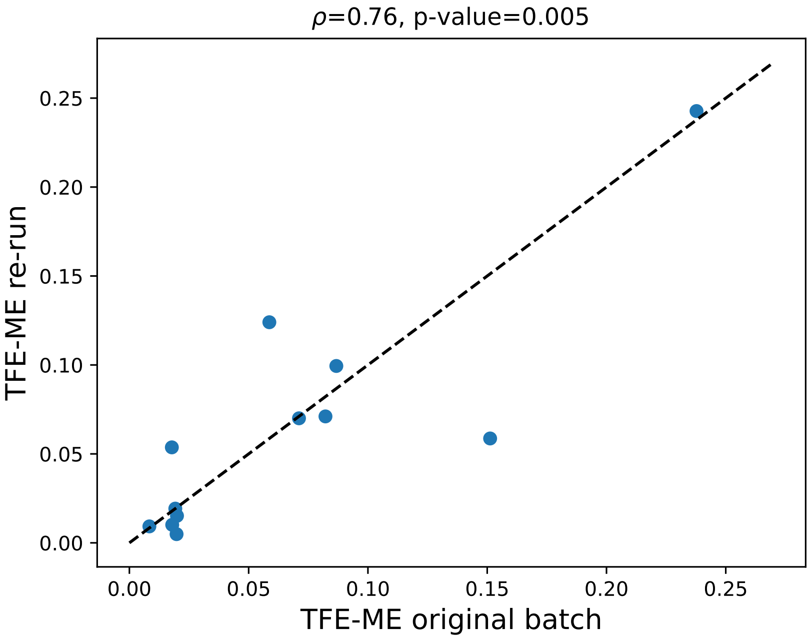

Figure S7: We generated pre-operative cfDNA methylation profiles from the same 12 CRLM patients (dots) twice on two separate sequencing batches. The $x-$axis represents the *TFE-ME* of these patients as used in this paper and the $y-$axis the *TFE-ME* estimated from the re-run.

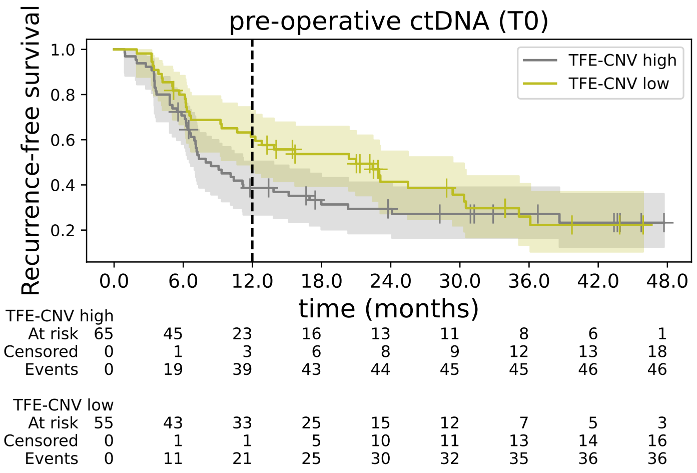

(a)

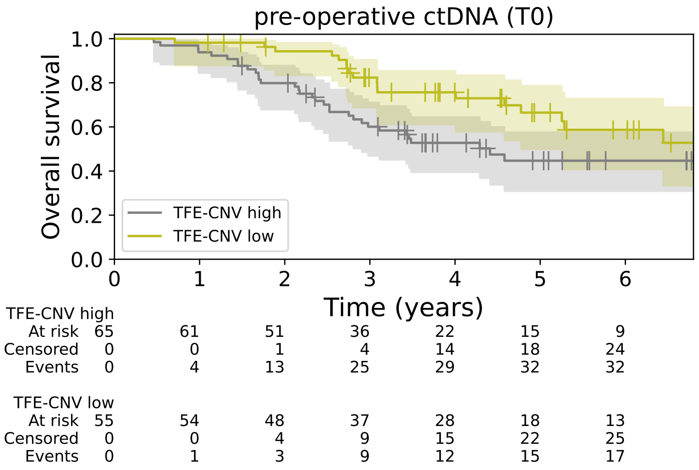

(b)

Figure S8: **(a)** Kaplan-Meier curves for 1-year RFS for patients that have detectable ctDNA (gray) versus undetectable (yellow) pre-operatively (T0) according to the *TFE-CNV*. The 95% confi- dence intervals of each curve are designated by the shaded areas. **(b)** As in a), but for OS.

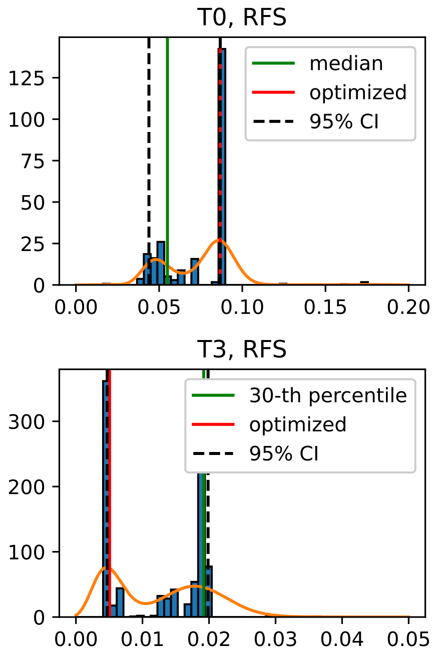

Figure S9: Distribution of the optimal cut-off determined by maximally selected rank statistics across 1,000 bootstraps for the pre-operative (T0, top) and post-operative (T3, bottom) *TFE-ME*. The first column shows the distribution for 1-year RFS and the second for OS. The optimal cut-off as determined on the entire dataset is shown as a red line, the 95% confidence interval based on the bootstraps is denoted by black dashed lines, and our choice of cut-off is shown as a green line.

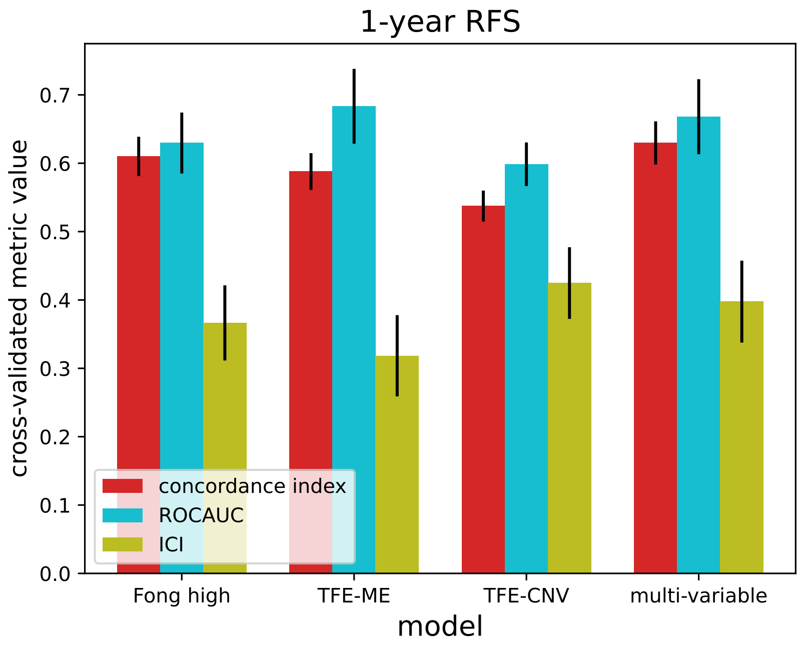

Figure S10: Performance of different models for 1-year RFS evaluated using the concordance index (CI), the area under the ROC curve (ROCAUC) and the integrated calibration index (ICI). Higher CI and ROCAUC and lower ICI correspond to better performance.

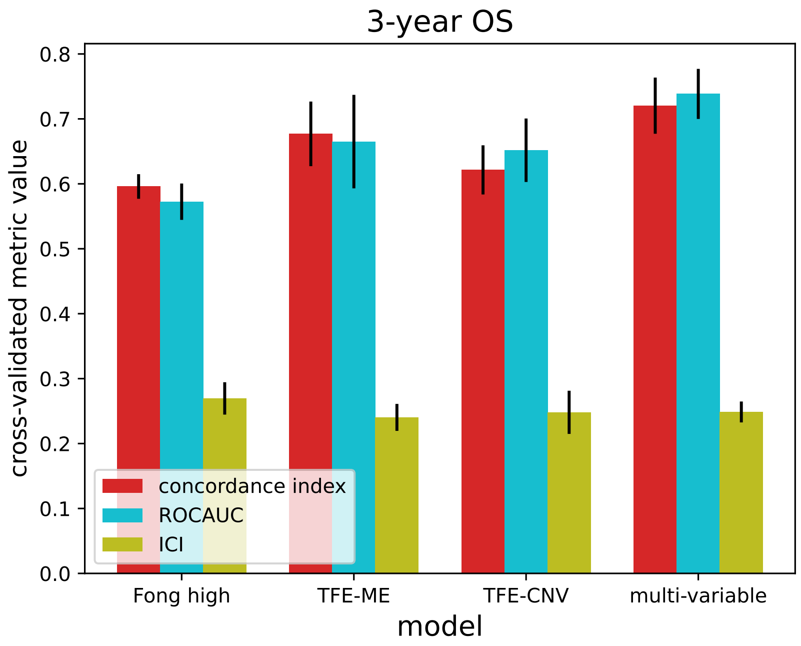

Figure S11: Performance of different models for 3-year OS evaluated using the concordance index (CI), the area under the ROC curve (ROCAUC) and the integrated calibration index (ICI). Higher CI and ROCAUC and lower ICI correspond to better performance.

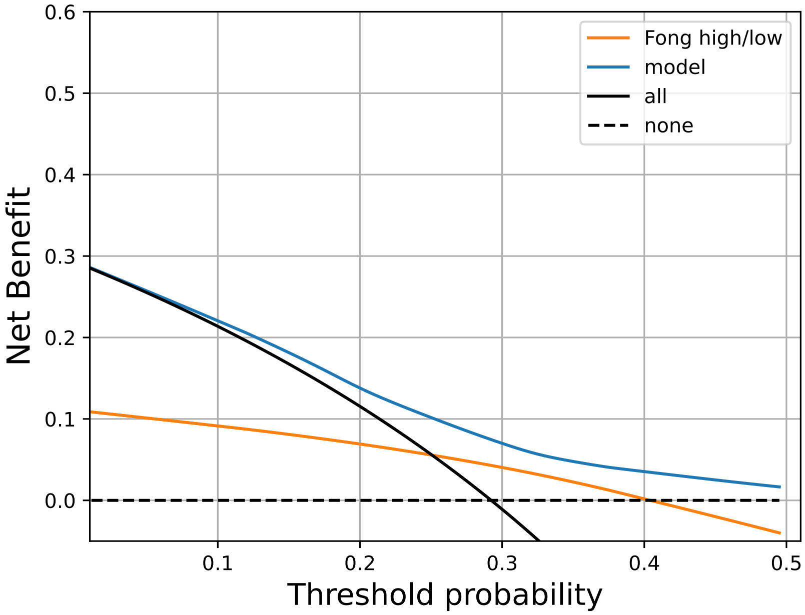

Figure S12: Net benefit analysis (decision curves [9]) for administering neo-adjuvant chemotherapy along a range of clinically relevant thresholds based on risks estimated using a model including pre-operative *TFE-ME* and clinical parameters. The ”treat all” and ”treat none” strategies are shown in solid and dashed black lines respectively while the decision curve of the model is in blue and of the Fong score in orange.

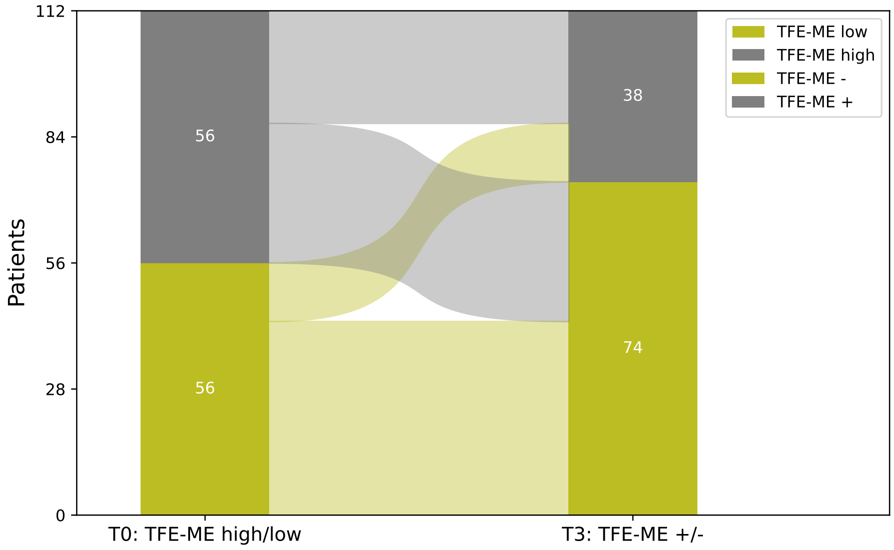

Figure S13: Conversion rates of the TFE-ME from high/low at T0 to positive/negative at T3 for 112 patients with both samples available.

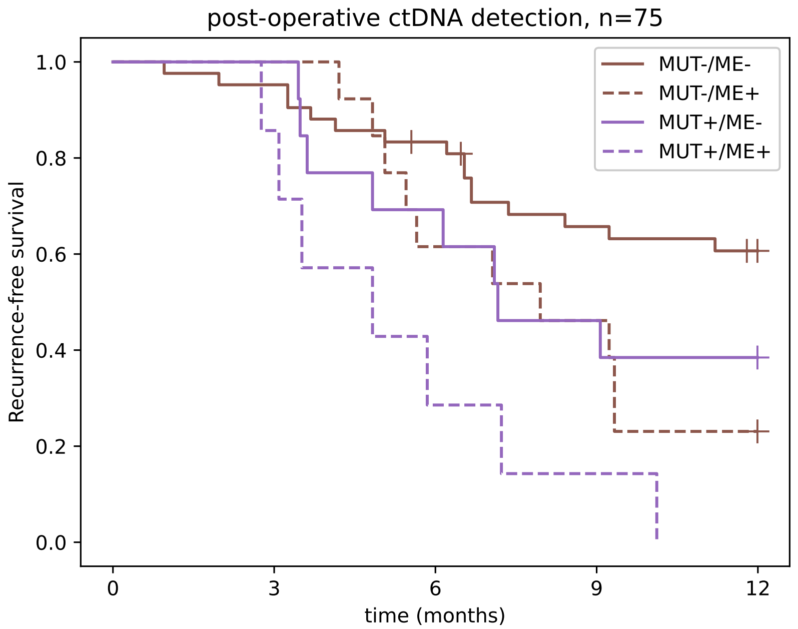

Figure S14: Kaplan-Meier curves for RFS of 75 patients with an assessable Oncomine mutation detected at T0. Patients are stratified by the status of ctDNA positivity at T3 based on mutations (mutation negative: brown, mutation positive: purple) and methylation (methylation negative: solid line, methylation positive: dashed line).

Supplementary Tables

Table S1: Clinical and pathological characteristics of the methylation cohort compared to the rest of the patients in the MIRACLE study.

	**Methylation cohort (n=120)**	**Remaining cohort (n=68)**	**p-value**
**One-year recurrence**			0.88
Yes	60	33	
No	60	35	
**Median OS (months)**	63.4	Not reached	0.46
**Median age (years)**	66.0	68.0	0.17
**Sex**			0.62
Male	81	49	
Female	39	19	
**Inclusion center**			**0.00063**
Albert Schweitzer	8	12	
Amphia	23	24	
Erasmus MC	68	19	
IJsselland	21	13	
**DFI from primary to CRLM**			0.42
$>$ 1 year	37	25	
$<$ 1 year	83	43	
**Lymph node status primary**			0.75
N0	46	24	
N+	72	43	
**Number CRLMs**			0.097
$\geq$ 2	63	27	
$<$ 2	57	41	
**Pre-operative CEA**			0.36
High	77	33	
Low	30	18	
**Diameter largest CRLM**			0.18
$\geq$ 5 cm	13	3	
$<$ 5 cm	107	64	
**Resection margin**			0.75
R0	109	61	
R1	8	3	
**Risk group Fong**			1.0
Low	87	48	
High	32	18	
**Diagnosis CRLM**			0.76
Synchronous	43	26	
Metachronous	77	42	
**Location of primary tumor**			0.92
Left-sided	52	28	
Rectum	39	24	
Right-sided	29	16	
**KRAS status primary tumor**			0.82
Wild-type	25	20	
Mutant	20	13	
Unknown	75	43	
**BRAF status primary tumor**			0.86
Wild-type	44	23	
Mutant	1	1	
Unknown	75	44	

Table S2: Genomic coordinates (GRCh38) of 28 CpG islands previously reported as cfDNA methylation markers for CRC.

**Chromosome**	**Start**	**End**	**Publication**
chr17	77372605	77374424	[2]
chr16	28062495	28063975	[2]
chr20	38805562	38806949	[2]
chr1	110673621	110675315	[2]
chr2	29115116	29116043	[2, 4]
chr8	96159576	96159794	[2]
chr10	17228430	17230618	[2]
chr3	14810321	14811587	[7]
chr13	93226991	93228624	[7]
chr8	71843547	71844432	[7]
chr17	77373541	77373616	[3]
chr9	129620114	129620228	[3]
chr11	105610527	105610633	[3]
chr8	71843764	71843886	[3]
chr6	133241282	133241378	[3]
chr17	45262101	45262200	[3]
chr2	181457503	181457579	[3]
chr5	115816849	115816958	[11]
chr20	62475984	62476044	[13]
chr4	153789072	153789132	[13]
chr12	24949057	24949159	[12]
chr6	392035	392145	[12]
chr7	50304270	50304365	[12]
chr13	110307146	110307206	[6]
chr2	74514400	74514460	[6]
chr2	181457336	181457396	[6]
chr6	72621213	72623381	[4]
chr9	129620152	129620725	[4]

Table S3: False Discovery Rates of the association between clinical, biological, and technical variables and the *TFE-ME* and *TFE-CNV*. p-values generated from the same test were jointly corrected for multiple testing using the Benjamini-Hochberg procedure.

**Variable**	**Effect**	**Test**	**FDR TFE-ME**	**FDR TFE-CNV**
CpG reads	technical	Spearman ρ	0.82	0.069
% CpG reads	technical	Spearman ρ	0.80	0.17
cfDNA concentration	technical	Spearman ρ	0.98	0.59
Mapping	technical	Spearman ρ	0.20	0.26
Sequencing batch	technical	Kruskal-Wallis	0.39	0.26
Inclusion center	technical	Kruskal-Wallis	0.39	0.51
Age	biological	Spearman ρ	0.87	0.61
Sex	biological	Mann-Whitney U	0.46	0.37
**APC mutant**	biological	Mann-Whitney U	**0.009**	**0.031**
**TP53 mutant**	biological	Mann-Whitney U	0.13	**0.031**
KRAS mutant	biological	Mann-Whitney U	0.50	0.89
PIK3CA mutant	biological	Mann-Whitney U	0.18	0.25
1-year DFI from primary to CRLM	clinical	Mann-Whitney U	0.33	0.89
Lymph node positivity	clinical	Mann-Whitney U	0.80	0.92
Number CRLMs >2	clinical	Mann-Whitney U	0.14	0.29
**CEA >200**	clinical	Mann-Whitney U	0.13	**0.049**
**Diameter largest CRLM** > 5	clinical	Mann-Whitney U	**0.009**	**0.0007**
Resection margin	clinical	Mann-Whitney U	0.50	0.78
**Risk group Fong**	clinical	Mann-Whitney U	0.08	**0.031**
Synchronous/Metachronous	clinical	Mann-Whitney U	0.99	0.78

Table S4: Multi-variable Cox model for predicting 1-year RFS and OS using the pre-operative *TFE-CNV*.

**Variable**	**RFS**			**OS**		
	HR	95% CI	p-value	HR	95% CI	p-value
Age (standardized)	1.10	[0.81, 1.49]	0.53	1.38	[0.99, 1.93]	0.056
Sex male	0.86	[0.50, 1.51]	0.61	0.65	[0.35, 1.19]	0.16
TFE-CNV > 0	1.55	[0.87, 2.76]	0.14	1.68	[0.89, 3.19]	0.11
Fong high risk	**2.03**	**[1.13, 3.66]**	**0.018**	1.66	[0.88, 3.11]	0.12
Right-sided tumor (ref. left-sided)	0.75	[0.39, 1.44]	0.39	1.12	[0.55, 2.31]	0.75
Rectal tumor (ref. left-sided)	**0.51**	**[0.27, 0.98]**	**0.04**	1.01	[0.50, 2.05]	0.97
Metachronous metastasis	0.61	[0.33, 1.13]	0.11	0.72	[0.38, 1.37]	0.32
KRAS mutant	1.29	[0.73, 2.28]	0.37	1.46	[0.80, 2.66]	0.22

Table S5: Comparison of predefined and optimal cut-offs by time point and outcome.

**Time point**	**Our cut-off**	**Outcome**	**Optimal cut-off**	**95% CI**
T0	0.055	RFS	0.087	[0.044, 0.087]
		OS	0.038	[0.019, 0.135]
T3	0.019	RFS	0.005	[0.005, 0.020]
		OS	0.018	[0.005, 0.023]

Table S6: Generalizability of the median cut-off across hospitals.

**Outcome**	**Training set**	**Test set size**	**Cut-off**	**HR test data [95% CI]**	**Log-rank p-value**
RFS	EMC	52	0.064	2.7 [1.3, 5.5]	0.01
	Peripheral	68	0.051	2.4 [1.1, 5.5]	0.03
OS	EMC	52	0.064	2.3 [1.1, 4.9]	0.02
	Peripheral	68	0.051	1.5 [0.6, 3.6]	0.41

Table S7: Generalizability of the median cut-off across batches.

**Outcome**	**Training set**	**Test set size**	**Cut-off**	**HR test data [95% CI]**	**Log-rank p-value**
RFS	G1	63	0.071	2.3 [1.1,4.9]	0.02
	G2	57	0.049	3.1 [1.3, 7.5]	0.01
OS	G1	63	0.071	1.4 [0.7, 3.0]	0.39
	G2	57	0.049	4.7 [1.4, 16.0]	0.01

Table S8: Predictive performance of different models for RFS and OS evaluated using the concordance index (CI), the area under the ROC curve (ROCAUC) and the integrated calibration index (ICI). Higher CI and ROCAUC and lower ICI correspond to better performance.

**Outcome**	**Measurement**	**CI**	**1-year ROCAUC**	**1-year ICI**
RFS	Fong score	0.61 ± 0.03	0.63 ± 0.04	0.36 ± 0.05
	TFE-ME	0.59 ± 0.03	0.68 ± 0.05	0.32 ± 0.06
	TFE-CNV	0.54 ± 0.02	0.60 ± 0.03	0.42 ± 0.05
	multi-variable + *TFE-ME*	0.63 ± 0.03	0.67 ± 0.05	0.40 ± 0.06
**Outcome**	**Measurement**	**CI**	**3-year ROCAUC**	**3-year ICI**
OS	Fong score	0.60 ± 0.02	0.57 ± 0.03	0.27 ± 0.02
	TFE-ME	0.68 ± 0.05	0.67 ± 0.07	0.24 ± 0.02
	TFE-CNV	0.62 ± 0.04	0.65 ± 0.05	0.25 ± 0.03
	multi-variable + *TFE-ME*	0.72 ± 0.04	0.74 ± 0.04	0.25 ± 0.016

Table S9: Multi-variable analysis of 1-year recurrence using both post-operative methylation and mutation data (n=73).

Covariate	HR [95% CI]	p-value
**age (standardized)**	**1.70 [1.12, 2.58]**	**0.013226**
sex male	0.84 [0.38, 1.86]	0.668484
TFE-ME@T0 (cont.)	**1.36 [1.01, 1.84]**	**0.045381**
**Fong high risk**	**2.10 [1.05, 4.20]**	**0.036514**
Rightsided tumor (ref. left-sided)	1.06 [0.44, 2.53]	0.901711
Rectal tumor (ref. left-sided)	0.44 [0.19, 1.03]	0.059328
**metachronous metastasis**	**0.38 [0.16, 0.90]**	**0.027435**
***TFE-ME*@T3 (cut-off)**	**3.20 [1.52, 6.73]**	**0.002191**
mutation positive@T3	2.00 [0.98, 4.08]	0.055211
KRAS mutant	1.46 [0.70, 3.08]	0.313764

Table S10: Multi-variable analysis of 1-year recurrence using the continuous *TFE-ME* at T0 and T3.

Covariate	HR [95% CI]	p-value
age	1.20 [0.87, 1.65]	0.27
sex male	0.85 [0.47, 1.55]	0.60
**Fong high risk**	**2.77 [1.55, 4.95]**	**0.0006**
Right-sided tumor (ref. left-sided)	0.70 [0.36, 1.36]	0.29
Rectal tumor (ref. left-sided)	0.50 [0.25, 1.00]	0.050
metachronous metastasis	0.64 [0.34, 1.20]	0.16
***TFE-ME*@T0 (cont.)**	**1.22 [1.04, 1.43]**	**0.014**
**log(*TFE-ME*@T3)**	**1.31 [1.03, 1.68]**	**0.03**
KRAS mutant	1.45 [0.79, 2.67]	0.23

Table S11: Reduced list of markers needed to measure *TFE-ME*. The markers are listed in descending order based on their importance for the prediction according to the model.

**Chromosome**	**Start**	**End**	**Coefficient**	**Promoter**	**Enhancer**
chr13	25468555	25469348	0.153971		
chr10	122163335	122164027	0.100277	TACC2	
chr13	36431458	36432316	0.0802	CCNA1	
chr12	81077790	81078340	0.070177	ACSS3	ACSS3
chr7	20776619	20776920	0.059766		
chr8	96159577	96159794	0.057183		
chr8	96160406	96161652	0.055575	GDF6	GDF6
chr11	70662223	70662512	0.051815		
chr9	707021	707420	0.05131		KANK1
chr8	17026854	17027830	0.046288	MICU3	
chr1	246788912	246789209	0.045962		
chr6	38715173	38715489	0.043316	DNAH8	GLO1
chr9	135075264	135076020	0.041605	OLFM1	
chr8	48735143	48735429	0.041163	EFCAB1	EFCAB1
chr2	236507447	236507739	0.040944	IQCA1	IQCA1
chr20	46251251	46251649	0.038936		
chr13	112062811	112063107	0.037912		
chr14	76138656	76139020	0.036265		
chr7	38630839	38631561	0.035345	AMPH	
chr19	36605055	36605639	0.034015	ZNF529, ZNF382	ZNF529
chr5	38556120	38557461	0.033861	LIFR	LIFR, LIFR-AS1
chr2	114662151	114662684	0.027947		DPP10
chr7	27220482	27220848	0.024375		
chr8	56157128	56157488	0.023446		
chr7	128104142	128104502	0.022877		SND1, LRRC4
chr7	98869400	98870435	0.01991	TMEM130	
chr2	153872431	153872815	0.019294		
chr10	101236277	101236889	0.018758		
chr17	78450564	78450788	-0.01827		DNAH17
chr6	134176398	134176618	0.017476		SGK1
chr18	7116853	7118242	0.017426	LAMA1	LAMA1
chr6	100447897	100448186	-0.01588		
chr7	38975943	38976329	0.014737		
chr5	45695292	45696408	0.012179	HCN1	
chr16	31569238	31569702	0.010242		
chr4	173509235	173509710	0.008476		HAND2-AS1
chr3	181726621	181727212	0.007996		
chr10	44292857	44293121	-0.0062		
chr4	110618072	110618327	0.005569		PITX2
chr2	63053899	63054212	0.003926		
chr15	64823814	64824368	0.002603		PLEKHO2, RBPMS2
chr2	80322453	80322673	0.001423		
chr15	45377772	45378831	0.000625	GATM	SPATA5L1

Table S12: Performance of reduced marker models across hospitals and batches.

	**Train on EMC**	**Train on peripheral**	**Train on batches 1-3**	**Train on batches 4-7**
Training R^2	0.670	0.754	0.704	0.780
Test R^2	0.775	0.748	0.693	0.637
Markers selected	24	30	67	35
Spearman ρ	0.887	0.905	0.846	0.842
Univariable HR (OS)	1.68	1.25	1.58	1.31

References

[1] Bárbara Andrade Barbosa, Saskia D van Asten, Ji Won Oh, Arantza Farina-Sarasqueta, Joanne Verheij, Frederike Dijk, Hanneke W M van Laarhoven, Bauke Ylstra, Juan J Garcia Vallejo, Mark A van de Wiel, and Yongsoo Kim. Bayesian log-normal deconvolution for enhanced in silico microdissection of bulk gene expression data. *Nat. Commun.*, 12(1):6106, October 2021.

[2] Manny D Bacolod, Aashiq H Mirza, Jianmin Huang, Sarah F Giardina, Philip B Feinberg, Steven A Soper, and Francis Barany. Application of multiplex bisulfite PCR-ligase detection reaction-real-time quantitative PCR assay in interrogating bioinformatically identified, blood-based methylation markers for colorectal cancer. *J. Mol. Diagn.*, 22(7):885–900, July 2020.

[3] Ludovic Barault, Alessio Amatu, Giulia Siravegna, Agostino Ponzetti, Sebastian Moran, Andrea Cassingena, Benedetta Mussolin, Chiara Falcomatà, Alexandra M Binder, Carmen Cristiano, Daniele Oddo, Simonetta Guarrera, Carlotta Cancelliere, Sara Bustreo, Katia Bencardino, Sean Maden, Alice Vanzati, Patrizia Zavattari, Giuseppe Matullo, Mauro Truini, William M Grady, Patrizia Racca, Karin B Michels, Salvatore Siena, Manel Esteller, Alberto Bardelli, Andrea Sartore-Bianchi, and Federica Di Nicolantonio. Discovery of methylated circulating DNA biomarkers for comprehensive non-invasive monitoring of treatment response in metastatic colorectal cancer. *Gut*, 67(11):1995–2005, November 2018.

[4] Sarah Østrup Jensen, Nadia Øgaard, Mai-Britt Worm Ørntoft, Mads Heilskov Rasmussen, Jesper Bertram Bramsen, Helle Kristensen, Peter Mouritzen, Mogens Rørbæk Madsen, Anders Husted Madsen, Kåre Gotschalck Sunesen, Lene Hjerrild Iversen, Søren Laurberg, Ib Jarle Christensen, Hans Jørgen Nielsen, and Claus Lindbjerg Andersen. Novel DNA methylation biomarkers show high sensitivity and specificity for blood-based detection of colorectal cancer-a clinical biomarker discovery and validation study. *Clin. Epigenetics*, 11(1):158, November 2019.

[5] Berthold Lausen and Martin Schumacher. Maximally selected rank statistics. *Biometrics*, 48(1):73, March 1992.

[6] Xianglin Liu, Jialing Wen, Chujun Li, Hui Wang, Jianping Wang, and Hongzhi Zou. High-yield methylation markers for stool-based detection of colorectal cancer. *Dig. Dis. Sci.*, 65(6):1710–1719, June 2020.

[7] Takafumi Nakano, Seiichiro Takao, Katsushi Dairaku, Naoki Uno, Siew-Kee Amanda Low, Masahiro Hashimoto, Yasuo Tsuda, Yuichi Hisamatsu, Takeo Toshima, Yusuke Yonemura, Takaaki Masuda, Ken Eto, Toru Ikegami, Yosuke Fukunaga, Atsushi Niida, Satoshi Nagayama, and Koshi

Mimori. Implementable assay for monitoring minimum residual disease after radical treatment for colorectal cancer. *Cancer Sci.*, 115(6):1989–2001, June 2024.

[8] Mark D Robinson and Gordon K Smyth. Moderated statistical tests for assessing differences in tag abundance. *Bioinformatics*, 23(21):2881–2887, November 2007.

[9] Andrew J Vickers and Elena B Elkin. Decision curve analysis: a novel method for evaluating prediction models. *Medical Decision Making*, 26(6):565–574, 2006.

[10] Andrew J Vickers, Ben van Calster, and Ewout W Steyerberg. A simple, step-by-step guide to interpreting decision curve analysis. *Diagnostic and prognostic research*, 3(1):18, 2019.

[11] Keishi Yamashita, Mina Waraya, Myoung Sook Kim, David Sidransky, Natsuya Katada, Takeo Sato, Takatoshi Nakamura, and Masahiko Watanabe. Detection of methylated CDO1 in plasma of colorectal cancer; a PCR study. *PLoS One*, 9(12):e113546, December 2014.

[12] Graeme P Young, Erin L Symonds, Hans Jørgen Nielsen, Linnea Ferm, Ib J Christensen, Evelien Dekker, Manon van der Vlugt, Rosalie C Mallant-Hent, Nicky Boulter, Betty Yu, Michelle Chan, Gregor Tevz, Lawrence C LaPointe, and Susanne K Pedersen. Evaluation of a panel of tumor-specific differentially-methylated DNA regions in IRF4, IKZF1 and BCAT1 for blood-based detection of colorectal cancer. *Clin. Epigenetics*, 13(1):14, January 2021.

[13] Xie Zhang, Yu-Fei Song, Hong-Na Lu, Dan-Ping Wang, Xue-Song Zhang, Shi-Liang Huang, Bei-Lei Sun, and Zhi-Gang Huang. Combined detection of plasma GATA5 and SFRP2 methylation is a valid noninvasive biomarker for colorectal cancer and adenomas. *World J. Gastroenterol.*, 21(9):2629–2637, March 2015.
